# Recent Advances in Mediator and Proton Relay Usage in Molecular Catalysis

**DOI:** 10.1002/advs.202515364

**Published:** 2026-01-08

**Authors:** Yu‐Lin Chi, Wei‐Shuo Chuang, Jing‐Ke Lin, Yu‐Heng Wang

**Affiliations:** ^1^ Department of Chemistry National Tsing Hua University 101, Sec 2, Kuang‐Fu Rd. Hsinchu 30013 Taiwan

**Keywords:** catalytic Tafel plots, molecular catalysis, electron–proton transfer mediator, redox mediator, proton relay, linear free energy relationship (LFER)

## Abstract

Electron–proton transfer mediators (EPTMs), redox mediators (RMs), and proton relays (PRs) have been incorporated into diverse homogeneous molecular catalysts to enhance catalytic efficiencies in the reactions of energy‐related small molecules (e.g., CO_2_, O_2_,H_2_O, and N_2_) and organic compounds. However, the benefits of using EPTMs, RMs, and PRs in molecular catalysis have not been comprehensively or quantitatively benchmarked. This review highlights the latest developments in this field, focusing on the kinetics, thermodynamics, and selectivities of multiple proton–electron redox processes, as well as their corresponding advantages. The reaction mechanisms, linear free energy relationships, and catalytic Tafel plots are considered to provide a solid foundation for evaluating these catalytic systems and the strategic development of molecular catalysts. These efforts are expected to advance the application of molecular catalysis in energy conversion and sustainability.

## Graphical abstract

The use of electron–proton transfer mediators, redox mediators, and proton relays in synergy with molecular catalysts in energy‐related small‐molecule and organic transformations is discussed, with a focus on recent advances. Linear free‐energy relationship and catalytic Tafel plots provide a systematic foundation for benchmarking these catalytic systems and the rational design of molecular catalysts.

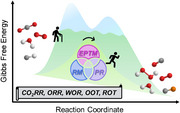

## 1 Introduction

The global energy crisis and climate change represent some of the most important challenges currently faced by humankind. Anthropogenic activities, including the extensive use of fossil fuels as a primary energy source, have increased the levels of atmospheric greenhouse gases, thereby intensifying the natural greenhouse effect and contributing to climate change.^[^ ^1^, ^2^, ^3^ ^]^ Given the depletion of fossil fuel reserves and increasingly apparent environmental consequences of their combustion, sustainable and renewable energy sources are highly sought after, and the corresponding research is gaining importance.^[^ ^4^, ^5^ ^]^ In particular, considerable attention has been paid to the development of catalytic processes for the efficient conversion of energy‐related small molecules (e.g., H_2_O, CO_2_, N_2_, and O_2_), along with catalytic pathways enabling sustainable organic transformations.^[^ ^6^, ^7^, ^8^ ^]^

Molecular catalysis is an effective means of addressing these concerns and achieving green and sustainable chemical transformations. Molecular catalysts often provide atomic‐level mechanistic insights, facilitating the rational design and optimization of catalysts tailored to renewable energy–relevant chemical transformations.^[^ ^9^, ^10^, ^11^, ^12^, ^13^ ^]^ By harnessing the well‐defined chemical structures of molecular catalysts, one can finely control reaction pathways and fine‐tune the reactivities of the small molecules central to renewable energy. This tuning can enable the modulation of the selectivity and efficiency of related processes, including the oxidation of H_2_O^[^ ^14^, ^15^, ^16^, ^17^ ^]^ and NH_3_,^[^ ^18^, ^19^, ^20^ ^]^ reduction of CO_2_^[^ ^21^, ^22^, ^23^ ^]^ and O_2_,^[^ ^24^, ^25^, ^26^ ^]^ N_2_ fixation,^[^ ^27^, ^28^, ^29^ ^]^ and organic transformations.^[^ ^30^, ^31^, ^32^ ^]^ These merits highlight the potential of molecular catalysts for practical energy conversion applications.

The advantages of molecular catalysts include their tunable electronic and steric properties, which can be altered through the modification of the primary and secondary coordination spheres (PCSs and SCSs, respectively).^[^ ^33^, ^34^, ^35^, ^36^, ^37^, ^38^, ^39^, ^40^ ^]^ In a coordination complex, the PCS comprises ligands directly attached to the metal center. For non‐hemilabile ligands, the coordinating atoms are firmly bound and cannot be readily replaced by the Lewis bases present in the environment. Nevertheless, the coordinating atoms in the PCS may still be involved in catalysis for hemilabile ligands, as their binding to the metal center is dynamic and partially disengaged in the solution state.^[^ ^41^ ^]^ The SCS comprises substituents that are not directly coordinated to the metal center but engage with the PCS (typically through non‐covalent interactions) and may influence the reactivities of molecular catalysts and stabilities of reaction intermediates.

The performances of molecular catalysts can be enhanced by modifying their SCSs through the incorporation of electron–proton transfer mediators (EPTMs),^[^ ^42^, ^43^, ^44^ ^]^ redox mediators (RMs),^[^ ^45^, ^46^ ^]^ and proton relays (PRs).^[^ ^47^, ^48^, ^49^, ^50^, ^51^, ^52^ ^]^ In the case of EPTMs, both electron transfer (ET) and proton transfer (PT) can be accelerated, and the catalytic reaction may proceed via concerted proton–electron transfer (CPET). RMs can facilitate the transport of electrons to or from the active catalytic sites, thereby enhancing the kinetics of multiredox reactions under mild conditions.^[^ ^42^, ^43^, ^44^, ^45^, ^46^, ^47^, ^48^, ^49^, ^52^, ^53^ ^]^ Additionally, strategically designed PRs oriented toward metal–substrate binding sites enable efficient proton shuttling in catalytic reactions involving multiproton transfer.^[^ ^42^, ^43^, ^44^, ^45^, ^46^, ^47^, ^48^, ^49^, ^51^, ^52^, ^54^ ^]^ Under these circumstances, the activation barriers of the reaction can decrease because of the avoided formation of high‐energy intermediates, which improves catalytic efficiency and potentially influences product selectivity.^[^ ^24^, ^26^, ^53^, ^55^, ^56^ ^]^ In addition to the covalent linkage of an RM or EPTM on the SCS of molecular catalysts, the use of a superstoichiometric RM or EPTM along with the catalyst is another strategy for examining the RM or EPTM effectiveness before starting the complicated synthesis of catalysts. These two approaches have distinct merits to improve reaction efficiency. For example, an RM covalently anchored on the molecular catalyst can accelerate intramolecular ET, while free RMs can promote outer‐sphere ET between the electrode and molecular catalyst under electrochemical conditions.^[^ ^57^, ^58^ ^]^

Although various EPTMs, RMs, and PRs have been used to promote the transformations of energy‐related small molecules and organic substrates, the related kinetic and thermodynamic benefits remain unclear. This review presents the recent advances in molecular catalysts integrated with EPTMs, RMs, and PRs, quantitatively examining their contributions to kinetics and thermodynamics based on log(rate)–driving force correlations, i.e., linear free‐energy relationship (LFER) analysis.^[^ ^59^, ^60^, ^61^ ^]^ This analysis provides valuable insights into catalytic performances in terms of kinetics (i.e., maximum turnover frequency (TOF_max_)) or catalytic rate constant (*k*_cat_)), and thermodynamics (overpotential (*η*)) or substrate bond dissociation energy (BDE)).

By highlighting recent innovations in catalyst design and enhancements in catalytic efficacy, this review illustrates how these molecular‐level strategies facilitate rational catalyst design and guide future research toward impactful and sustainable catalytic solutions. Ultimately, molecular catalysis is expected to help address the intertwined challenges of energy conversion and climate resilience, thereby supporting a transition to a more sustainable global energy future.

## 2 EPTMs, RMs, and PRs in Molecular Catalysis

**Scheme 1:**
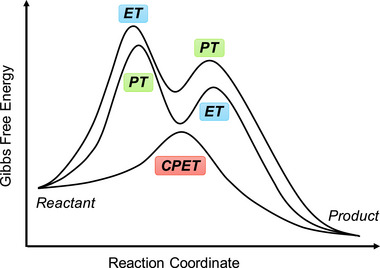
Schematic Gibbs free energy diagrams of proton transfer followed by electron transfer (PTET), electron transfer followed by proton transfer (ETPT), and concerted proton–electron transfer (CPET).

Homogeneous redox catalysis typically involves multiple proton–electron transfers in a single turnover, a process known as the stepwise proton‐coupled electron transfer (PCET) pathway (**Scheme** **1**).^[^ ^62^, ^63^, ^64^, ^65^ ^]^ This pathway comprises two main processes, namely ET followed by PT (ETPT) or vice versa (PTET). Either of these processes may encounter a kinetically unfavorable route because of the formation of short‐lived charged intermediates. In contrast, CPET involves the simultaneous transfer of an electron and a proton within a single reaction step, as opposed to the sequential transfer mentioned above (Scheme 1).^[^ ^66^, ^67^, ^68^ ^]^ This process may offer advantages over sequential ETPT or PTET, as it circumvents the formation of high‐energy intermediates and potentially reduces the activation energy. Consequently, effective methods of rendering redox reactions more efficient are highly sought after.

For a stepwise ETPT or PTET pathway, either the PT or ET can be the turnover‐limiting step (TLS). RM and PR utilization are expected to lower the kinetic energy barrier of ET and PT, respectively. If the activation energy of the TLS is notably lowered with the assistance of RMs or PRs, the reaction mechanism may change from ETPT or PTET to CPET because of the TLS change. EPTMs are capable of shuttling both PT and ET, and a CPET pathway becomes possible if PT and ET are concertedly promoted and occur. Collectively, RMs, PRs, and EPTMs have been utilized in molecular catalysis to promote kinetically favorable pathways and enhance catalytic activity.

### 2.1 EPTMs in Molecular Catalysis

**Scheme 2:**
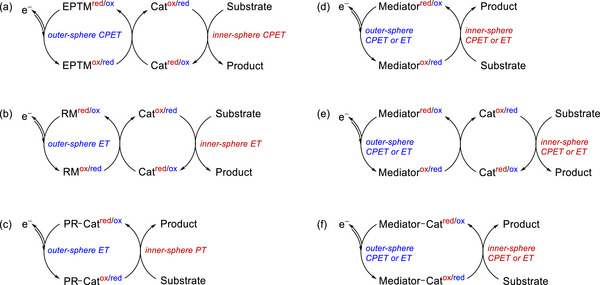
Schematic showing a) electron–proton transfer mediators (EPTMs), b) redox mediators (RMs), and c) proton relays (PRs) in molecular catalysis, as well as the utilization of d) solely EPTMs or RMs, e) self‐independent EPTMs or RMs with the molecular catalyst, and f) EPTMs or RMs covalently anchored to the molecular catalyst.

EPTMs are molecules that promote the concurrent movement of electrons and protons during chemical reactions (**Scheme** **2**a).^[^ ^42^, ^43^, ^44^ ^]^ Acting as transporters, these species enable a more efficient and often lower‐energy transfer of electrons and protons compared with scenarios where these transfers occur independently, such as ETPT or PTET pathways. Specifically, EPTMs may encourage the reaction to follow the CPET pathway with optimal energy efficiency, thereby enhancing reaction kinetics (i.e., increasing *k*_cat_ and TOF_max_).^[^ ^44^, ^45^ ^]^ Thus, one can circumvent energetic reaction pathways and thereby maintain catalyst longevity to achieve reasonable turnover numbers (TONs). For a multiple proton–electron transfer reaction, different selectivities can be achieved with the use of EPTMs, as the product distribution can presumably be modulated through control of PT and ET.^[^ ^46^, ^69^ ^]^

### 2.2 RMs in Molecular Catalysis

RMs are molecules that facilitate outer‐sphere ET between a catalyst and an electrode, changing the ET in the molecular catalyst from a heterogeneous to a homogeneous process (Scheme 2b).^[^ ^45^, ^46^ ^]^ The thus enhanced regeneration of catalytically active species results in improved catalytic performance.^[^ ^57^, ^58^ ^]^ RMs (or EPTMs) can also refer to redox‐active units in the SCS that facilitate intramolecular ET (or CPET) between them and substrate‐bound active sites and often enhance the efficiency of redox reactions.^[^ ^70^, ^71^ ^]^ Beyond this context, ligands containing RMs (or EPTMs) in the PCS can potentially act as redox reservoirs and participate in ET (or CPET) alongside the metal center in molecular metal complexes, in which case they are termed “redox non‐innocent ligands.”^[^ ^72^, ^73^, ^74^, ^75^, ^76^ ^]^ Importantly, ligand–metal cooperative redox events can help delocalize electron density and stabilize transient intermediates with high‐energy oxidizing/reducing states.

### 2.3 PRs in Molecular Catalysis

In biological frameworks such as enzymes, PRs correspond to molecules within a system where protons are effectively transferred from a distant location to the reaction center through a chain of hydrogen bonds.^[^ ^77^, ^78^ ^]^ This process relays the proton along the sequence, facilitating a (bio)chemical reaction by enabling rapid proton movement over distances exceeding the limitations of a single hydrogen bond. From the perspective of molecular catalysts, PRs are substituents located within a catalyst, generally dangling in the SCS and oriented toward the catalytically active center (Scheme 2c).^[^ ^47^, ^48^, ^49^, ^52^ ^]^ These substituents function as proton conduits, facilitating swift and targeted PT to/from the bound substrate and thereby promoting PT during reactions.

A PR should be carefully designed to ensure that it plays the expected role in a redox reaction. For a molecular catalyst, the dangling PR should have an appropriate p*K*_a_ matching that of the catalyst resting state. Balanced proton donation and acceptance, based on the reaction environment, must be considered, as the p*K*_a_ values of PRs and catalytically active centers are influenced by the medium.^[^ ^47^, ^64^, ^79^ ^]^ Furthermore, PRs should be positioned close to the catalytic center for effective PT. A well‐designed hydrogen‐bonded network ensures directional and efficient proton flow throughout the reaction. Importantly, the PR should resist oxidation/reduction and degradation over multiple catalytic cycles. To implement this concept in molecular catalysts, substituents containing acidic (i.e., carboxyl and sulfo) or basic (i.e., amino and pyridyl) groups have been introduced into the SCSs of metal complexes to promote the water oxidation.^[^ ^50^ ^]^

### 2.4 Strategies for Implementing Mediators and PRs in Molecular Catalysis

Homogeneous redox catalysis with EPTMs or RMs generally relies on the utilization of i) solely EPTMs or RMs (Scheme 2d), ii) self‐dependent EPTMs or RMs combined with the molecular catalyst (Scheme 2e), or iii) EPTMs or RMs covalently anchored to the molecular catalyst (Scheme 2f).^[^ ^42^, ^44^, ^80^, ^81^ ^]^ In case (i), EPTMs or RMs are themselves regarded as catalysts and can directly promote the conversion of reactants into products in the absence of a molecular co‐catalyst. In case (ii), the reaction mixture is commonly supplemented with a superstoichiometric quantity of mediators to ensure that the reaction kinetics are not limited by the mass transfer rate of mediators.^[^ ^82^ ^]^ Although this strategy is convenient and easy to realize, the solubility of a mediator depends on its identity and the reaction medium, and excess mediators in the solution can hinder in situ experiments aimed at investigating the reaction intermediates.^[^ ^83^, ^84^ ^]^

The coupling of a mediator with a molecular catalyst via covalent linkages is another strategy for engaging the mediator to shuttle protons and electrons during the redox process. Conceptually, this approach is similar to PR installation in the SCS of molecular catalysts.^[^ ^47^, ^48^, ^49^, ^52^ ^]^ A prominent advantage of mediator‐integrated molecular catalysts is that intramolecular PT and/or ET are presumably more effective than the intermolecular processes promoted by exogenous mediators. Moreover, the absence of superstoichiometric exogenous mediators eliminates potential interference during the in situ monitoring of reaction intermediates, thereby facilitating the elucidation of reaction mechanisms. Despite the above merits, mediator‐containing hybrid catalysts may be challenging to synthesize and design.

The redox potential of the mediator and the distance between the catalytically active center and mediator (similar to the PR case) may require fine‐tuning to optimize PT and/or ET kinetics. For example, it may be necessary to locate tethered mediators or PRs near the catalytic center in an appropriate orientation to facilitate intramolecular ET and PT.^[^ ^48^, ^85^, ^86^ ^]^ The stabilities of mediators or PRs as substituents in molecular catalysts also require a comprehensive examination to confirm that the observed enhancement in redox catalysis is not due to heterogeneous catalysts (e.g., nanoparticles or metal oxides) generated by catalyst degradation.^[^ ^87^, ^88^ ^]^ Thus, in comparison with the use of exogenous mediators, rational catalyst design regarding catalyst–mediator dyads remains challenging.

## 3 Molecular Catalysis: Definition and Metrics

Performance assessments from kinetic and thermodynamic perspectives are important for understanding and enhancing the efficiencies of molecular catalysts.^[^ ^50^, ^61^, ^89^ ^]^ The parameters commonly used to evaluate molecular catalysts include the Faradaic efficiency (FE), quantum yield (QY) or apparent quantum yield (AQY), TON, TOF_max_, and *η*, which are discussed below.

### 3.1 FE

FE is a direct measure of the efficacy of an electrochemical reaction, corresponding to the proportion of the total cumulative charge (*Q*_tot_, C) used to generate the desired product (*Q*_prod_, C) (Equation 1).^[^ ^90^, ^91^ ^]^ The determination of *Q*_prod_ (C) requires knowing the quantity of the obtained product (*N*_prod_, mol), which can be determined using different instrumental techniques. Consequently, FE can be obtained by estimating *Q*_prod_ using Faraday's law of electrolysis (Equation 2, where *n*_c_ is the number of electrons per turnover, e.g., four for O_2_/H_2_O, and *F* is Faraday's constant, i.e., 96485 C mol^−1^).

(1) $$FE\left(\%\right)=\frac{Q_{\mathrm{prod}}}{Q_{\mathrm{tot}}}\;\times \;100$$

(2) $$\mathrm{FE}\left(\%\right)=\frac{N_{\mathrm{prod}}n_{c}F}{Q_{\mathrm{tot}}}\;\times \;100$$

### 3.2 QY or AQY

In photocatalysis, QY denotes the proportion of absorbed photons (*N*_pa_) that transform a substrate molecule into the target product (*N*_prod_) during a light‐driven chemical reaction (Equation 3).^[^ ^92^ ^]^ Depending on the wavelength of the employed light, QY can be estimated using different chemical and physical actinometries.^[^ ^92^, ^93^, ^94^, ^95^ ^]^ Owing to the difficulties in quantifying the photons absorbed by the photocatalyst, the QY associated with photocatalysis can be derived by replacing the measured *N*_pa_ with the estimated number of incident photons, in which case the resulting parameter is denoted as AQY (Equation 4).^[^ ^89^, ^96^, ^97^ ^]^ In Equation (4), *N*_A_ is Avogadro's constant (6.02 × 10^23^ mol^−1^), *h* is Planck's constant (6.626 × 10^−34^ J s^−1^), *c* is the speed of light in vacuum (3 × 10^8^ m s^−1^), *P* is the power density of the monochromatic incident light (W m^−2^), *S* is the irradiation area (m^2^), *λ*_i_ is the wavelength of the incident monochromatic light, and *t* is the duration of the exposure to incident light (*s*)

(3) $$\mathrm{QY}\left(\Phi_{\mathrm{QY}},\%\right)=\frac{N_{\mathrm{prod}}}{N_{\mathrm{pa}}}\;\times \;100$$

(4) $$\mathrm{AQY}\left(\Phi_{\mathrm{AQY}},\%\right)=\frac{N_{\mathrm{prod}}n_{c}N_{A}\;\times \;\mathrm{hc}}{\mathrm{PS}\lambda_{i}t}\;\times \;100$$

In the ideal scenario (*Φ*_QY(AQY)_ = 1), every absorbed photon results in the formation of one molecule of the desired product. However, *Φ*_QY(AQY)_ is typically less than unity because of light scattering, reflection, and other non‐productive processes.^[^ ^98^, ^99^ ^]^ Additionally, *Φ*_QY(AQY)_ values markedly exceeding unity generally imply radical chain reactions.^[^ ^100^ ^]^ Overall, *Φ*_QY(AQY)_ represents the effectiveness of the photocatalytic process, corresponding to the number of reaction turnovers triggered by each absorbed photon.

### 3.3 TON

In homogeneous catalysis, the TON is defined as the number of substrate molecules converted into product molecules (*N*_prod_) per unit of catalyst (*N*_cat_), as shown in Equation (5).^[^ ^101^, ^102^, ^103^ ^]^ Given that time is not factored into the calculation, the TON is a dimensionless metric and may therefore not accurately reflect the kinetic properties of the catalyst. For example, a catalyst with a slow but steady activity can achieve a high TON. However, the long‐term durability of a catalyst does not necessarily imply that it exhibits optimal kinetic performance.^[^ ^50^, ^101^, ^102^ ^]^

(5) $$\mathrm{TON}=N_{\mathrm{prod}}/N_{\mathrm{cat}}$$

### 3.4 TOF

The TOF represents the TON relative to the reaction duration (*t*), i.e., the number of substrate molecules converted into product molecules per catalyst molecule within a given timeframe (Equation 6).^[^ ^101^, ^102^, ^103^ ^]^ The TOF is a uniform criterion for assessing catalyst kinetics, enabling the reactivity evaluations of various dissolved molecular catalysts under different reaction conditions. However, this metric provides limited information regarding catalyst durability and should therefore be used with caution. Notably, catalysts exhibiting higher TOFs may degrade within a short timeframe and, therefore, exhibit low long‐term performance and be poorly suited for practical applications.^[^ ^50^, ^101^, ^102^ ^]^

(6) $$\mathrm{TOF}=N_{\mathrm{prod}}/\left(N_{\mathrm{cat}}\times t\right)=\mathrm{TON}/t$$

For homogeneous electrocatalysts, the TOF_max_ can be calculated from cyclic voltammetry (CV) data (Equations 7 and 8):^[^ ^101^, ^104^ ^]^

(7) $$i_{c}=n_{c}\mathrm{FA}C_{\mathrm{cat}}\sqrt{D_{\mathrm{cat}}k_{\mathrm{obs}}}$$

(8) $$\frac{i_{c}}{i_{p}}=\frac{2.24n_{c}}{{n_{p}}^{3/2}}\sqrt{\frac{\mathrm{RT}k_{\mathrm{obs}}}{\mathrm{Fv}}}$$

In Equation (7), *i*_c_ is the catalytic current (plateau‐shaped wave or scan rate‐independent current (A)),^[^ ^104^ ^]^ *A* is the surface area of the working electrode (cm^2^)*, C*_cat_ is the catalyst concentration (m), *D*_cat_ is the diffusion coefficient of the catalyst (cm^2^ s^−1^), and *k*_obs_ is the observed rate constant (s^−1^) identical to the TOF_max_ value.^[^ ^101^, ^105^ ^]^ Equation (8) can be considered a variant of Equation (7) and is commonly used when the measurement of *A* or *D*_cat_ is experimentally challenging. Three parameters of Equation (8) need to be independently measured, namely the peak current of the catalyst (*i*_p_, A), the electron transfer number of the catalyst in the absence of the substrate (*n*_p_), and the scan rate (*v*, V s^−1^).

In instances where an ideal S‐shaped CV curve cannot be obtained because of side phenomena such as substrate depletion, catalyst deactivation, and product inhibition, foot‐of‐the‐wave analysis (FOWA) offers an alternative for the determination of TOF_max_ (Equation 9).^[^ ^101^, ^104^, ^106^ ^]^ FOWA primarily targets the “foot” region of the catalytic wave, a zone where intrinsic kinetic behavior is exhibited with minimal interference from side effects. In Equation (9), *E*_CAT_ is the catalytic potential, which is discussed in Section 3.5.

(9) $$\frac{i_{c}}{i_{p}}=\frac{2.24n_{c}\sqrt{\frac{\mathrm{RT}}{\mathrm{Fv}}k_{\mathrm{obs}}}}{1+\exp\left[\frac{F}{\mathrm{RT}}\left(E-E_{\mathrm{CAT}}\right)\right]}\;$$

### 3.5 η

*η* describes the additional energy beyond the thermodynamic equilibrium potential required for a reaction to occur at a measurable rate under particular conditions.^[^ ^61^, ^107^ ^]^ For molecular electrocatalysts, *η* can be determined as the difference between the thermodynamic potential (*E*_rxn_) and catalytic potential (*E*_CAT_) associated with the analyzed reaction (Equation 10).^[^ ^50^, ^61^, ^107^ ^]^ *η* reflects the influence of the solvent and reaction conditions on the thermodynamic properties of the electrochemical reaction (*E*_rxn_), as well as the catalytic potential of molecular catalysts (*E*_CAT_).

(10) $$\eta =|E_{\mathrm{rxn}}-E_{\mathrm{CAT}}|$$

Using *η* as a normalized metric, one can benchmark catalyst performances across different studies, even if the reaction conditions are not identical. However, the *η* values reported in different studies should be compared with caution, as various potentials have been employed to represent *E*_CAT_, including the half‐wave potential of the catalyst (*E*_1/2_), potential at the half‐maximum of *i*_c_ (*E*_cat/2_), potential corresponding to the emergence of *i*_c_ (*E*_onset_), anodic potential of the irreversible wave related to the catalyst (*E*_a_), and applied potential of controlled‐potential electrolysis (*E*_CPE_). Depending on the choice of *E*_CAT_, *η* may exhibit discrepancies of more than 100 mV.^[^ ^107^ ^]^ Therefore, when comparing the *η* values reported in different studies, one should ascertain whether the definition of *η* is consistent across these studies.

### 3.6 Log(TOF)−η Analysis and Catalytic Tafel Plot

**Figure 1:**
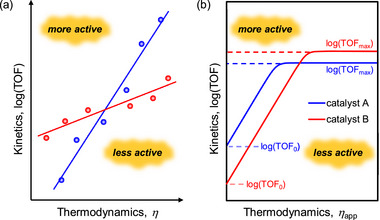
a) Representative log[turnover frequency (TOF)]−overpotential (*η*) correlation for different series of molecular catalysts (i.e., red and blue dots). b) Representative catalytic Tafel plot for different molecular catalysts.

The value of *η* required to achieve the TOF demonstrated by a specific catalyst is a crucial measure of its efficacy. An LFER can be constructed to examine the proportional influence of *η* on log(TOF). Given that the TOF and *η* are considered standardized metrics, LFER analysis can independently benchmark the effectiveness of catalysts, assessing catalyst performance through a 2D approach (**Figure** **1**a). For catalysts positioned in the upper left corner of Figure 1a, a minimal *η* is required to achieve a high catalytic rate, which is characteristic of high‐performance catalysts.

LFER analysis often reveals a trade‐off relationship between kinetics and thermodynamics for homogeneous catalysts. A higher TOF generally results in a higher *η*, and vice versa.^[^ ^61^, ^107^, ^108^ ^]^ Nonetheless, the strategic design of catalysts in the SCS and manipulation of reaction conditions may help circumvent this conventional scaling relationship.^[^ ^109^, ^110^, ^111^ ^]^ Importantly, the same series of catalysts is expected to align with the same linear trend of log(TOF)/*η* because the catalysts possess identical TLSs.^[^ ^112^, ^113^ ^]^ Thus, caution should be exercised when benchmarking catalyst performance exclusively based on LFER analysis without insights into the reaction mechanism, as these catalysts may feature different TLSs.^[^ ^114^, ^115^ ^]^

In addition, catalytic Tafel plots have emerged as an alternative for describing the relationship between catalytic activity and energetic driving force in molecular electrocatalysis.^[^ ^60^, ^61^, ^116^ ^]^ Unlike traditional heterogeneous Tafel analysis, which relates current density to applied overpotential (*η*_app_), (homogeneous) catalytic Tafel plots describe how the observed TOF of molecular catalysts depends on *η*_app_ (Figure 1b). This method provides a unified way of benchmarking catalysts that may differ in structure, mechanism, and reaction conditions.

The mathematic basis of Tafel plot analysis is shown in Equation (11), where TOF_max_ is *η*_app_−independent TOF. By extrapolating the log(TOF) curve to *η*_app_ = 0 V, one can ascertain the intrinsic catalytic activity of a molecular catalyst, TOF_0_ (Figure 1b). At a low driving force, the catalytic rate increases exponentially with *η*_app_, reflecting the fraction of catalyst molecules activated through reduction or oxidation. In this regime, the slope of the plot approximates the Nernstian value, signifying that ET governs the population of the active catalyst. As *η*_app_ is increased further, the plot transitions into a plateau region where the catalyst is almost completely present in its active redox state. Here, the rate becomes independent of ET and approaches TOF_max_​, which corresponds to the *k*_cat_ of the TLS (Equation 12). The resulting curve is sigmoidal in shape, capturing the potential‐dependent activation and rate‐limiting chemical step.

(11) $$\mathrm{TOF}=\frac{\mathrm{TO}F_{\max}}{1+\exp[\frac{F}{\mathrm{RT}}\;(|E_{\mathrm{rxn}}-E_{\mathrm{CAT}}|-\eta_{\mathrm{app}}\;)]}$$

(12) $$\mathrm{TO}F_{\max}=k_{\mathrm{obs}}=k_{\mathrm{cat}}\left[\mathrm{substrate}\right]$$

## 4 Applications of EPTMs, RMs, and PRs in Molecular Catalysis

In this section, the benefits of using EPTMs, RMs, and PRs in the molecular redox catalyst–promoted transformations of small energy‐related molecules and organic reactions are discussed from the perspectives of kinetics, thermodynamics, and selectivity. The involvement of these co‐catalysts in the reaction mechanism is also discussed.

### 4.1 CO2 Reduction Reaction (CO2RR)

#### 4.1.1 EPTMs in the Molecular Catalyst–Promoted CO2RR

**Figure 2:**
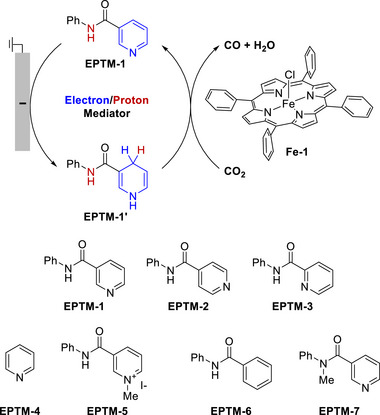
CO_2_ reduction reaction (CO_2_RR) catalyzed by Fe‐1 in the presence of different mediators (EPTM‐*n*, *n* = 1–7). Reproduced with permission.^[^ ^117^ ^]^ Copyright 2020, American Chemical Society.

**Figure 3:**
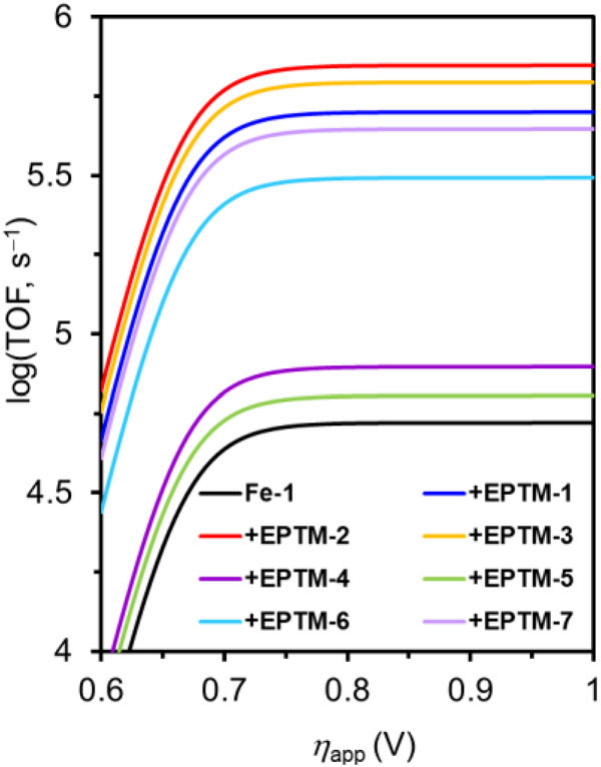
Catalytic Tafel plots obtained for **Fe‐1** alone and upon the addition of 1 equiv. **EPTM‐*n*** (***n*** = **1**–**7**). Reproduced with permission.^[^ ^117^ ^]^ Copyright 2020, American Chemical Society.

Chang et al. (2020) used nicotinamide adenine dinucleotide (NAD^+^/NADH) analogs (**Figure** **2**, EPTM‐1–7) as EPTMs with Fe tetraphenylporphyrin (Fe(TPP), Fe‐1) as a catalyst to promote the electrochemical CO_2_RR.^[^ ^117^ ^]^ In the presence of phenol (PhOH) as a proton source, the ECEC (E: electron transfer step, C: chemical step) behavior of EPTM‐1–3 was revealed by CV studies (Figure 2, EPTM‐1/EPTM‐1′). The importance of a 2H^+^/2e^−^ mediator in electrochemical CO_2_RR was evidenced by catalytic performance comparison, with EPTM‐1–3 being most effective (**Table** **1**). EPTM‐4 and EPTM‐5 were unable to mediate a 2H^+^/2e^−^ process, affording TOFs lower than those of the other EPTMs by around one order of magnitude. EPTM‐6 and EPTM‐7 served as proton‐ and electron‐only mediators, respectively, and their effectiveness in the electrochemical CO_2_RR was lower than that of EPTM‐1–3. This outcome suggested the crucial role of amide and pyridine substituents in the EPTM. The incorporation of EPTM‐1–7 into the system did not compromise the selectivity for CO, which remained the primary product, and had only a minimal effect on the FE (Table 1). The synergy between Fe‐1 and these NADH‐type mediators was further assessed using a catalytic Tafel plot, which revealed that at *η* ≥ 700 mV, the catalytic activity (i.e., TOF_max_) exceeded that observed for Fe‐1 alone ≈15‐fold (Table 1, entry 1 vs entries 2−4 and **Figure** **3**).

**Table 1:** Performances of Fe‐1 and Fe‐1/EPTM‐*n* (*n* = 1–7) systems in the electrochemical CO_2_RR.^[^ ^117^ ^]^

Catalyst/EPTM	Charge [C]^a)^	FE_CO_ [%]^b)^	*j*_CO_ [mA cm^−2^]^c)^	TOF_max_ [s^−1^]^d)^	*η* [V]
**Fe‐1**	41.8	93	0.67	5.24 × 10^4^	0.660
**Fe‐1/EPTM‐1**	119.2	86	2.07	5.00 × 10^5^	0.659
**Fe‐1/EPTM‐2**	141.0	108	2.45	7.01 × 10^5^	0.658
**Fe‐1/EPTM‐3**	132.7	87	2.31	6.23 × 10^5^	0.659
**Fe‐1/EPTM‐4**	50.4	94	0.82	7.85 × 10^4^	0.659
**Fe‐1/EPTM‐5**	46.0	92	0.74	6.39 × 10^4^	0.658
**Fe‐1/EPTM‐6**	94.5	104	1.63	3.10 × 10^5^	0.660
**Fe‐1/EPTM‐7**	112.5	100	1.95	4.44 × 10^5^	0.659

^a)^ Controlled‐potential electrolysis (CPE) experiments were performed for 0.5 mm **Fe‐1** in the presence and absence of 0.5 mm synthetic NADH analogs at −2.4 V vs Fc/Fc^+^ in 0.1 M tetrabutylammonium hexafluorophosphate (NBu_4_PF_6_) containing 500 mM phenol under a CO_2_ atmosphere for 16 h;

^b)^ Determined using gas chromatography with flame ionization detection and a known calibration curve. Faradaic efficiencies (FEs) were estimated to have an error of ±10%;

^c)^ Determined by averaging the observed current density during each 16 h electrolysis experiment and multiplying by FE_CO_;

^d)^ Estimated from the average *j*_CO_ during each electrolysis cycle.

**Figure 4:**
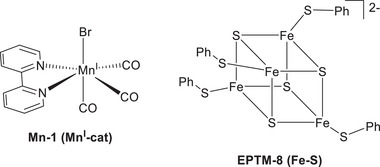
Mn complex **(Mn‐1**) and **EPTM‐8** used to investigate the electrochemical CO_2_RR.^[^ ^118^ ^]^

Mougel et al. reported a notable example highlighting the importance of mediator design, using a Mn complex to promote the electrochemical CO_2_RR (**Figure** **4**, **Mn‐1** and **EPTM‐8**).^[^ ^118^ ^]^ The bond dissociation free energy (BDFE) matching between the catalyst (Mn^I^‐H, 67.3–65.8 kcal mol^−1^) and EPTM ([Fe‐S]H, 63.5 kcal mol^−1^) was pivotal for optimizing reaction selectivity (FE_HCOOH_: 92%) and TOF (∼20 s^−1^). When the BDFE of the EPTM exceeded that of Mn^I^‐H, CO was the only product (**Table** **2**). This observation implies that [Mn^I^‐H], which could only be formed under the conditions of BDFE_cat‐H_ > BDFE_EPTM‐H_, served as the key species for the selective production of formate from CO_2_RR via the hydride transfer pathway.

**Table 2:** Performances of **Mn‐1** and **Mn‐1/EPTM‐8** systems in the electrochemical CO_2_RR.^[^ ^118^ ^]^

Conditions	*E*_CPE_ (V vs Fc^+/0^)	FE_CO_ / FE_HCOOH_ [%]	TOF [s^−1^]	*η* [V]
**Mn‐1** ^a)^	−1.85	91/0	–	0.22
**EPTM‐8** ^b)^	−1.85	0/30	–	0.22
**Mn‐1 + EPTM‐8 (1:1)** ^c)^	−1.85	9/87	–	0.22
**Mn‐1 + EPTM‐8 (1:2)** ^d)^	−1.85	6/92	20.3 ± 2	0.22‐0.34
**Mn‐1 + EPTM‐8 (1:2)** ^d)^	−1.70	6.7/91	0.46	0.07‐0.19
**Mn‐1 + EPTM‐8 (1:2)** ^d)^	−1.65	3/78	0.4	0.02‐0.14
**Mn‐1 + EPTM‐8 (1:2)** ^d)^	−2.05	32/56	–	0.42‐0.54

^a)^ 1 mM catalyst;

^b)^ 2 mM EPTM;

^c)^ 1 mM catalyst, 1.0 mM EPTM;

^d)^ 1 mM catalyst, 2 mM EPTM. General condition: 1 mM solutions of catalyst in MeCN containing 0.1 M NBu_4_PF_6_ as supporting electrolyte under 1 atm CO_2_ and in the presence of 0.2 m 2,2,2‐trifluoroethanol (TFE).

**Figure 5:**
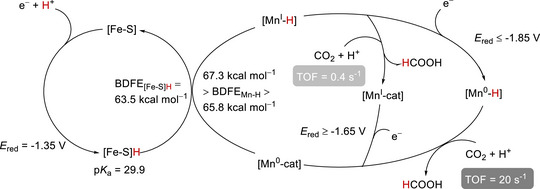
Proposed mechanism for **EPTM‐8**‐mediated **Mn‐1** formation and subsequent hydride transfer to CO_2_.^[^ ^118^ ^]^ Copyright 2022, Springer Nature.

A plausible catalytic cycle is depicted in **Figure** **5**. A single‐electron reduction and PT at a terminal Fe‐S‐Ph unit afford the activated form of **EPTM‐8** ([Fe‐S]H), and CPET between [Fe‐S]H and [Mn^0^‐cat] yields [Mn^I^‐H] and suppresses the formation of an off‐cycle dimeric Mn species. Subsequently, Mn^I^‐H is further reduced to [Mn^0^‐H], which then mediates the transfer of hydride to CO_2_, resulting in the production of HCOOH. This reductive pathway to [Mn^0^H] is thermodynamically more favorable than the direct formation of Mn^I^‐H via **Mn‐1** protonation in the absence of **EPTM‐8**. This work highlights the effectiveness of EPTM‐assisted strategies in advancing the electrochemical CO_2_RR.

#### 4.1.2 RMs in the Molecular Catalyst–Promoted CO2RR

**Figure 6:**
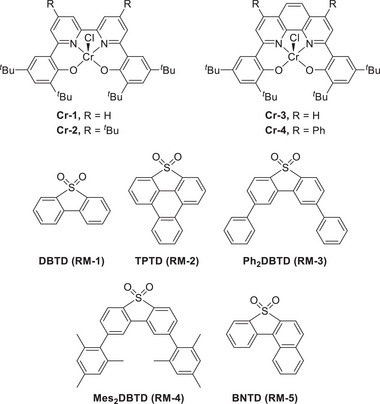
Cr complexes (Cr‐*n*, *n* = 1–4) and RMs (RM‐*n*, *n* = 1–5) used to investigate the electrochemical CO_2_RR.^[^ ^120^, ^121^, ^122^ ^]^

Machan et al. presented a cooperative electrocatalytic method for the conversion of CO_2_ into CO using a molecular Cr complex (Cr‐1) and dibenzothiophene‐5,5‐dioxide (RM‐1) as an RM (**Figure** **6**).^[^ ^119^ ^]^ The reduction potential of RM‐1 is important for the success of this co‐catalysis, as it must be lower than that of Cr‐1. TOFs of up to 65 s^−1^ were achieved at *η* = 410 mV using PhOH as a proton donor (**Table** **3**). In control experiments, no CO production was detected, and no coproducts (i.e., CO or CO_3_^2−^) were observed for either Cr‐1 or RM‐1 alone. The inner‐sphere ET from the bound RM‐1 to the di‐CO_2_ adduct of Cr‐1, ([Cr(CO_2_CO_2_(RM‐1)]^2−^), was facilitated by through‐space electronic conjugation (TSEC), helping RM‐1 effectively mediate the electrocatalytic CO_2_RR.

**Table 3:** Performances of **Cr‐*n*** (***n*** = **1**–**4**) and **Cr‐*n*** (***n*** = **1**–**4**)/**RM‐*n*** (***n*** = **1**–**5**) systems in the electrochemical CO_2_RR.^[^ ^120^, ^121^, ^122^ ^]^

Entry	Condition	Potential (V vs Fc^+^/Fc)	FE_CO_ [%]	TOF_CPE_ [s^−1^]	*η* [V]
1‐A	**Cr‐1** + PhOH^a^ ^)^	−2.30	111 ± 14	7.12	0.11
1‐A'	**Cr‐1** + PhOH^d)^	−2.30	95 ± 8	9.29	0.16
1‐A"	**Cr‐1** + PhOH^j)^	−2.30	111 ± 14	7.12	0.11
1‐b	**Cr‐1** + **RM‐1** ^f)^	−2.30	91 ± 10	36.8	0.69
1‐B	**Cr‐1** + PhOH + **RM‐1** ^b)^	−2.30	102 ± 12	65.3	0.41
1‐B'	**Cr‐1** + PhOH + **RM‐1** ^g)^	−2.30	102 ± 14	65.3	0.41
1‐C	**Cr‐1** + PhOH + **RM‐2** ^c)^	−2.25	98 ± 17	74.5	0.35
1‐D	**Cr‐1** + PhOH + **RM‐4** ^c)^	−2.30	98 ± 6	64	0.4
1‐E	**Cr‐1** + PhOH + **RM‐3** ^d)^	−2.20	100 ± 2	69.3	0.28
2‐A	**Cr‐2** + PhOH^)^ ^d)^	−2.30	95 ± 8	9.29	0.16
2‐B	**Cr‐2** + PhOH + **RM‐1** ^c)^	−2.30	109 ± 9	163	0.41
2‐C	**Cr‐2** + PhOH + **RM‐2** ^c)^	−2.25	97 ± 6	208	0.35
2‐D	**Cr‐2** + PhOH + **RM‐4** ^c)^	−2.30	98 ± 4	149	0.4
2‐E	**Cr‐2** + PhOH + **RM‐3** ^c)^	−2.20	97 ± 5	194	0.28
2‐F	**Cr‐2** + PhOH + **RM‐5** ^c)^	−2.20	100 ± 2	63.4	0.12
3‐A	**Cr‐3** + PhOH^)^ ^e)^	−2.30	101 ± 3	4.9	0.12
3‐B	**Cr‐3** + PhOH + **RM‐1** ^i)^	−2.30	94 ± 7	56.3	0.41
3‐E	**Cr‐3** + PhOH + **RM‐3** ^c)^	−2.20	102 ± 3	126	0.28
3‐F	**Cr‐3** + PhOH + **RM‐5** ^i)^	−2.20	103 ± 5	328	0.12
4‐A	**Cr‐4** + PhOH^h)^	−2.10	91 ± 3	0.24	0.09
4‐B	**Cr‐4** + PhOH + **RM‐1** ^i)^	−2.30	77 ± 2	27.4	0.41
4‐F	**Cr‐4** + PhOH + **RM‐5** ^i)^	−2.20	97 ± 5	34	0.12

^a^ ^)^0.5 mM catalyst, 0.6 M PhOH;

^b)^ 0.5 mM catalyst, 2.5 mM RM, 0.6 M PhOH;

^c)^ 0.1 mM catalyst, 0.5 mM RM, 0.12 M PhOH;

^d)^ 0.1 mM catalyst, 0.12 M PhOH ;

^e)^ 0.5 mM catalyst, 1.0 M PhOH ;

^f)^ 1.0 mM catalyst, 2.5 mM RM ;

^g)^ 1.0 mM catalyst, 2.5 mM RM, 0.6 mM PhOH ;

^h)^ 0.5 mM catalyst, 0.8 mM PhOH ;

^i)^ 0.1 mM catalyst, 0.5 mM RM, 1.0 M PhOH;

^j)^ 0.1 mM catalyst, 0.6 M PhOH.

**Figure 7:**
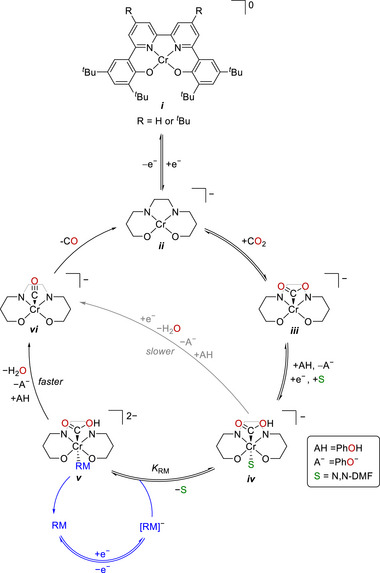
Catalytic cycle proposed for the electrochemical CO_2_RR promoted by Cr catalysts and RMs. Reproduced under terms of the CC‐BY‐NC license.^[^ ^120^ ^]^ Copyright 2022, Royal Society of Chemistry.

In 2022, the same group rationally designed catalysts (Cr‐2) and RMs (RM‐2–4) to circumvent the trade‐off between the TOF and *η* through the influence of TSEC and pancake bonding (PB) interactions between the Cr catalysts and RMs (Figures 6 and 7).^[^ ^120^ ^]^ **Figure** **7** shows the catalytic cycle proposed for the electrochemical CO_2_RR catalyzed by Cr‐1 and Cr‐2 with RM‐1–4. The formation of *iv* from *i* was presumably the thermodynamically favored process. In the presence of RMs, the single‐electron‐reduced RM associated with species *iv* to generate an important intermediate, *v*. The C–OH bond cleavage of *v* was reported as the TLS, and [Cr–CO]^−^ (*vi*) was formed concurrently with the release of the neutral RM and H_2_O upon the incorporation of a second equivalent proton. Following the liberation of CO, *vi* transformed into a monoanionic four‐coordinate neutral Cr species (*ii*), and the catalytic cycle was completed. The impact of PB interactions became more prominent with the increasing number of phenyl groups in the RM. For example, a quantitative selectivity for CO at *η* = 280 mV along with a TOF of 194 s^−1^ was achieved using Cr‐2, 2,8‐diphenyldibenzothiophene‐5,5‐dioxide (RM‐3), and PhOH as the proton source (Table 3). This result indicates that the electron‐donating *tert*‐butyl substituents on the backbone bipyridine (bpy) motif improved the kinetics of the CO_2_RR.

Subsequently, a catalyst bearing a phenanthroline (phen) backbone (Figure 6, Cr‐3) was prepared to enhance PB interactions and more efficiently promote the CO_2_RR. The compensatory relationship between PB and dispersion interactions affected the formation of Cr−RM adducts. Consequently, a TOF of 126 s^−1^ was achieved at *η* = 280 mV in the presence of RM‐3 and PhOH (Table 3).^[^ ^121^ ^]^ The same group also demonstrated that the *E*_CAT_ of the CO_2_RR was correlated with the *E*_1/2_ of the RM (RM‐1 or RM‐5), and optimized performance was achieved when the *E*_1/2_ of the RM matched that of the Cr catalyst (Table 3).^[^ ^122^ ^]^ This finding implies that the redox potentials of the catalysts and RMs must be carefully considered in electrocatalysis.

**Figure 8:**
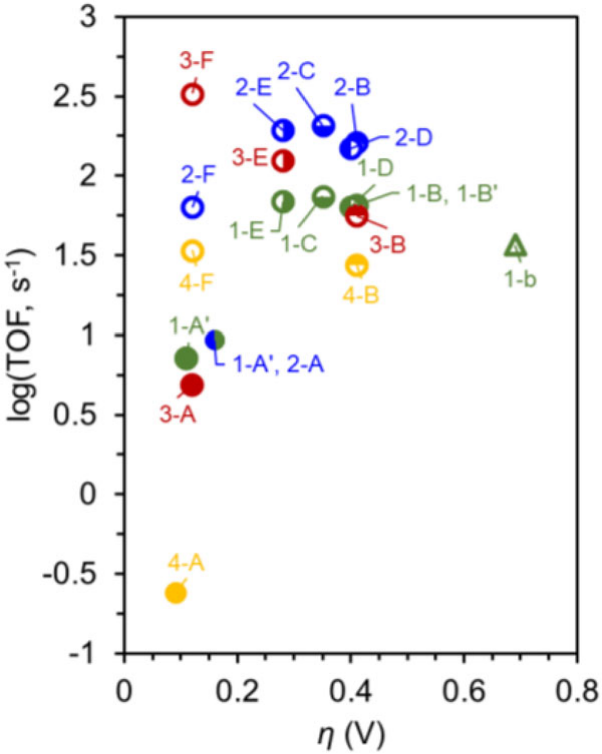
Results of linear free‐energy relationship (LFER) analysis obtained for **Cr‐*n*** (***n*** = **1**–**4**) and **Cr‐*n*** (***n*** = **1**–**4**)/**RM‐*n*** (***n*** = **1**–**5**) systems used to promote the electrochemical CO_2_RR.^[^ ^120^, ^121^, ^122^ ^]^

Figure 6 shows the log(TOF)−*η* plots of these Cr CO_2_RR catalysts with different RMs, revealing the dependence of the catalytic effectiveness on catalyst–RM cooperation. For example, Cr‐3 and Cr‐4 showed remarkable reaction kinetics in the presence of RM‐5 and PhOH, with *η* ≈ 0.1 V (data points: 2‐F, 3‐F, and 4‐F). The integration of RM‐3 with Cr‐1–3 using PhOH as the proton source gave comparable TOFs, and the reactions were operated at *η* ≈ 0.3 V (Table 3, entries 1‐E, 2‐E, and 3‐E). The catalytic turnover of the co‐catalytic systems exceeded those of catalyst‐only systems by several orders of magnitude at similar *η* values (**Figure** **8**, points 1‐A, 2‐A, 3‐A, and 4‐A). These results demonstrate that the interplay between RMs and catalysts is crucial for enhancing the efficacy of redox catalysis, suggesting that the consideration of tethered substituents and redox potentials is necessary to optimize molecular RMs.

**Figure 9:**
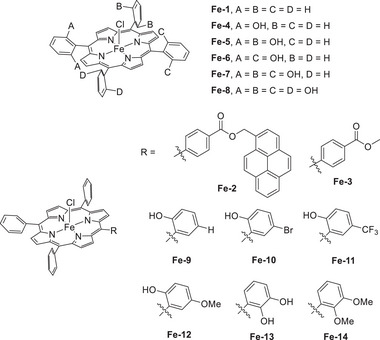
Fe(TPP) complexes, **Fe‐*n* (*n*** = **1**–**14**), used for the electrochemical CO_2_RR.^[^ ^123^, ^124^, ^125^, ^126^ ^]^

**Figure 10:**
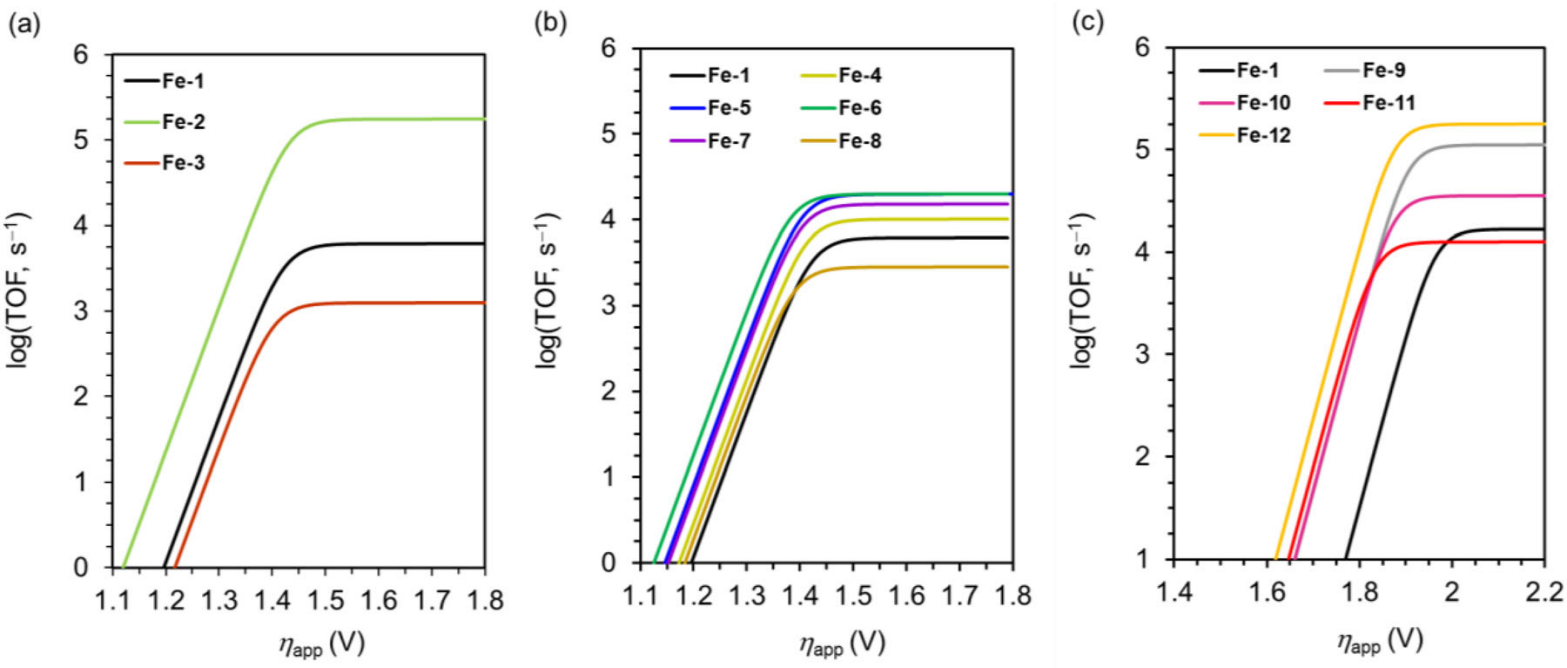
Tafel plots constructed for the electrochemical CO_2_RR catalyzed by different Fe(TPP) complexes: a) **Fe‐1**–**3**, b) **Fe‐1** and **Fe‐4**–**8**, and c) **Fe‐1**, and **Fe‐9–12**. Reproduced with permission.^[^ ^123^, ^124^, ^125^ ^]^ Copyright 2024, American Chemical Society.

Warren et al. (2024) used an Fe(TPP)–pyrene complex to promote the electrocatalytic CO_2_RR (**Figure** **9**, **Fe‐2**).^[^ ^123^ ^]^ Unlike **Fe‐3**, **Fe‐2** contains a redox‐active pyrenyl group in the TPP framework. This design enabled intramolecular ET from the pyrenyl group to the Fe metal center under electrochemical conditions and resulted in CO_2_‐to‐CO catalytic turnovers exceeding those of **Fe‐1** and **Fe‐3** 10‐ to 100‐fold (**Table** **4** and Figure 10a).^[^ ^123^ ^]^ The intramolecular ET rate was influenced by the proximity of the RM to the catalytic center. This insight suggests that caution must be exercised in the design of pendant RMs for the SCS of molecular catalysts.

**Table 4:** TOF and *η* values obtained for the electrochemical CO_2_RR catalyzed by Fe(TPP) complexes **Fe‐*n*** (***n* = 1**–**14**) in *N*,*N*‐dimethylformamide (DMF).^[^ ^123^, ^124^, ^125^, ^126^ ^]^

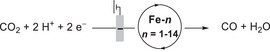			
Catalyst	TOF [s^−1^]	*η* [V]	Refs.
**Fe‐1** ^a)^	6.17 × 10^3^	1.42	[124]
**Fe‐2** ^b)^	1.78 × 10^5^	1.43	[123]
**Fe‐3** ^b)^	1.26 × 10^3^	1.40	[123]
**Fe‐4** ^a)^	1.02 × 10^4^	1.41	[124]
**Fe‐5** ^a)^	2.00 × 10^4^	1.401	[124]
**Fe‐6** ^a)^	2.00 × 10^4^	1.38	[124]
**Fe‐7** ^a)^	1.51 × 10^4^	1.40	[124]
**Fe‐8** ^a)^	2.82 × 10^3^	1.388	[124]
**Fe‐9** ^c)^	1.12 × 10^5^	1.90	[125]
**Fe‐10** ^c)^	3.55 × 10^4^	1.87	[125]
**Fe‐11** ^c)^	1.26 × 10^4^	1.83	[125]
**Fe‐12** ^c)^	1.78 × 10^5^	1.91	[125]
**Fe‐13** ^d)^	4.80 × 10^2^	−	[126]
**Fe‐14** ^d)^	1.00 × 10^2^	−	[126]

^a)^ TOF (= *k*_obs_) was calculated from the *i*_c_/*i*_p_ values extracted from the CV curves recorded under 1 atm CO_2_ with the addition of 100 m PhOH in MeCN;

^b)^ TOF (= *k*_obs_) was calculated from the *i*_c_/*i*_p_ values extracted from the CV curves recorded under 1 atm CO_2_ with the addition of 100 m PhOH in DMF;

^c)^ TOF (= *k*_obs_) was calculated using the FOWA of the CV curves recorded under 1 atm CO_2_ with the addition of 10 m PhOH in MeCN;

^d)^ TOF (= *k*_obs_) was calculated using the FOWA of the CV curves recorded under 1 atm CO_2_ with the addition of 10 mM PhOH in DMF.

#### 4.1.3 PRs in Molecular Catalyst–Promoted CO2RR

The CO_2_RR can also be promoted using catalysts with appropriately positioned PRs. Warren et al. developed Fe(TPP) catalysts bearing different numbers of *ortho*‐hydroxyl groups on their phenyl rings (Figure 9, **Fe‐4**–**8**).^[^ ^124^ ^]^ These groups increased the concentration of H^+^ around the Fe metal center, thereby accelerating the reaction, as evidenced by the increased catalytic currents and TOFs (Table 4). The corresponding catalytic Tafel plots showed that *cis*‐ and *trans*‐Fe(TPP)(OH)_4_ (**Fe‐5** and **Fe‐6**) exhibited higher activities than the other Fe(TPP)(OH)*_n_* catalysts (**Figure** **10**b, *n* = 0 (**Fe‐1**), 2 (**Fe‐4**), 6 (**Fe‐7**), and 8 (**Fe‐8**)). The volcano‐type correlation between the CO_2_RR activity and the number of attached hydroxyl groups revealed that the trade‐off between the TOF and *η* is an important factor to consider in catalyst design. The concentration of exogenous Brønsted acids (i.e., PhOH) and solvent type may influence the stability of the Fe–CO_2_ adduct and PT from the relay hydroxyl groups via hydrogen bonding. This influence should be further investigated to optimize ligand design and clarify the effect of the reaction medium on the outcome.

For Fe(TPP) catalysts bearing 2‐hydroxyphenyl groups with different electronic properties (Figure 9, **Fe‐9**–**12**), *k*_cat_ was correlated with catalyst p*K*_a_.^[^ ^125^ ^]^ For the catalyst with the lowest p*K*_a_ (**Fe‐11**), intramolecular PT was preferred. Instead, for the catalyst with the highest p*K*_a_ (**Fe‐12**), the stabilization of the reduced [Fe^I^–CO_2_] intermediate through hydrogen bonding played a crucial role, and intermolecular PT from PhOH was more favorable. The observed primary kinetic isotope effect indicated that protonation was the TLS for **Fe‐9–12**. The corresponding catalytic Tafel plot demonstrated that **Fe‐12**, bearing an electron‐donating methoxy substituent at the *para* position, afforded the fastest catalytic turnover (Figure 10c), which was attributed to the facile reprotonation of the internal hydroxyphenyl group of the [Fe^I^–CO_2_H] intermediate. **Fe‐13**, featuring a catechol unit ancillary to the TPP motif, exhibited a 1.7‐fold higher TOF than **Fe‐14** under the same reaction conditions (Table 4 and Figure 10c).^[^ ^126^ ^]^ Density functional theory analysis indicated that internal hydrogen bonding involving the catechol group of **Fe‐13** enhanced the stability of the [Fe^I^–CO_2_H] intermediate, thereby resulting in an internal PT more effective than that observed for **Fe‐14**.

**Figure 11:**
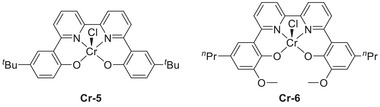
Cr complexes (Cr‐5 and Cr‐6) used to electrochemically catalyze the CO_2_RR.^[^ ^127^ ^]^

**Figure 12:**
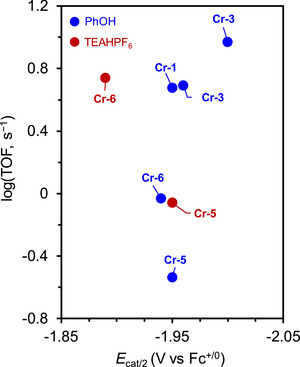
Log(TOF)–*E*_cat/2_ analysis of different Cr catalysts. Reproduced with permission.^[^ ^127^ ^]^ Copyright 2023, Royal Society of Chemistry.

Machan et al. (2024) reported a collaboration between Cr catalysts (**Figure** **11**, Cr‐5 and Cr‐6) and external Lewis acids (PhOH and triethylammonium hexafluorophosphate, TEAHPF_6_), revealing that the reaction rate was highest for Cr‐6/TEAHPF_6_ (**Table** **5**).^[^ ^127^ ^]^ The corresponding log(TOF)−*E*_cat/2_ analysis revealed that Cr‐6/TEAHPF_6_ demonstrated the best thermodynamic and kinetic performances for the electrochemical CO_2_RR (**Figure** **12**). The inverse relationship observed for this system was ascribed to the cationic acid stabilizing the negatively charged reaction intermediates with the assistance of the suspended methoxy group, which rendered the deprotonation of the [Cr–CO_2_H]^−^ intermediate less energetic.

**Table 5:** Performances of Cr‐*n* (*n* = 1–3, 5, and 6) catalysts in different proton source systems for the electrochemical CO_2_RR.^[^ ^127^ ^]^

Conditions	*E*_cat/2_ (V vs Fc^+/0^)	FE_CO_ [%]	TOF [s^−1^]	*η* [V]
**Cr‐1** + PhOH^a)^	−1.95	96 ± 8	4.75	0.11
**Cr‐2** + PhOH^b)^	−2.00	95 ± 8	9.29	0.16
**Cr‐3** + PhOH^c)^	−1.96	101 ± 3	4.9	0.12
**Cr‐5** + PhOH^d)^	−1.95	85 ± 3	0.29	0.09
**Cr‐5** + TEAHPF_6_ ^e)^	−1.95	83 ± 1	0.87	0.70
**Cr‐6** + PhOH^d)^	−1.94	99 ± 3	0.93	0.06
**Cr‐6** +TEAHPF_6_ ^f)^	−1.89	103 ± 5	5.47	0.66

^a)^ 0.58 mM catalyst, 0.62 M acid;

^b)^ 0.1 mM catalyst, 0.12 M acid;

^c)^ 0.5 mM catalyst, 1.0 M acid ;

^d)^ 0.75 mM catalyst, 1.5 M acid ;

^e)^ 0.5 mM catalyst, 20 mM acid ;

^f)^ 0.5 mM catalyst, 0.10 M acid, no electrolyte.

### 4.2 Oxygen Reduction Reaction (ORR)

#### 4.2.1 EPTMs in the Molecular Catalyst–Promoted ORR

**Figure 13:**
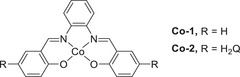
Co catalysts used for the oxygen reduction reaction (ORR): (a) **Co‐1** and (b) **Co‐2**.^[^ ^128^ ^]^

Mondal et al. (2023) synthesized a mononuclear Co complex (**Figure** **13**, **Co‐2**) bearing a salophen‐type Schiff‐base ligand (Sal) and covalently linked hydroquinone (H_2_Q).^[^ ^128^ ^]^ Unlike the bare (Sal)Co complex (Figure 13, **Co‐1**), which favored the 2H^+^/2e^−^ ORR affording H_2_O_2_, the complex with the appended H_2_Q units favored the 4H^+^/4e^−^ ORR affording H_2_O (**Table** **6**). CV and differential pulse voltammetry were used to examine the benzoquinone (BQ)/H_2_Q and Co^III/II^ redox couples, along with the *E*_onset_ of the catalytic ORR. **Co‐1** and **Co‐2** exhibited a catalytic wave ≈−1.1 V vs ferrocene (Fc)^+/0^; however, **Co‐2** displayed a markedly higher current than **Co‐1** under similar conditions (≈106 vs 45.8 µA). The higher catalytic current and different selectivities (Table 6) suggest that the attached H_2_Q moieties led to a change in the reaction mechanism.

**Table 6:** Performances of **Co‐1** and **Co‐2** systems in the (electro)chemical ORR.^[^ ^128^ ^]^

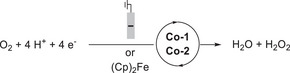			
Catalyst	Acid conc. [mM]	H_2_O_2_ [%]	H_2_O [%]
**Co‐1** ^a)^	−	45	−
**Co‐2** ^a)^	−	27	−
**Co‐1** ^b)^	30	97	3
**Co‐2** ^b)^	30	20	80

^a)^ Electrochemical ORR;

^b)^ Chemical ORR using decamethylferrocene (Cp*2Fe) as a reductant.

**Figure 14:**
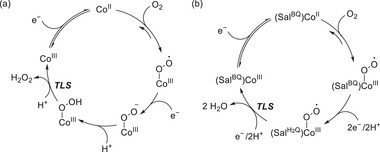
Catalytic cycles of the ORR promoted by (a) **Co‐1** and (b) **Co‐2**. Reproduced with permission.^[^ ^128^ ^]^ Copyright 2023, American Chemical Society.

At low and high acid concentrations (10 and 300 mM acetic acid, respectively), **Co‐2** exhibited a reactivity comparable with that of **Co‐1** in the presence of 10 and 2 equiv. exogenous BQ, respectively. Changes in acid concentration, which influence the reactivity of **Co‐1** and **Co‐2**, are indicative of a consequential change in the TLS. At a low acid concentration, the protonation of Co^III^−O_2_H, which liberates H_2_O_2_, could be the TLS (**Figure** **14**a); while the transfer of 2H^+^/1e^−^ to (Sal^H2Q^)Co−O_2_^•^ is proposed to be TLS for **Co‐2** (Figure 14b), as suggested by the slope of 92 mV dec^−1^ for the corresponding *E*_cat/2_ vs log[acetic acid (AcOH)] plot. At a high acid concentration (i.e., AcOH ≥ 100 mM), the step of cobalt superoxide formation serves as the TLS for both **Co‐1** and **Co‐2**.

**Figure 15:**
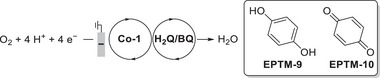
Electrochemical ORR catalyzed by Co‐1 in the presence of EPTM‐9 (0−40 mm).^[^ ^129^ ^]^ Conditions: 1.0 mm Co‐1, 0.3 m acetic acid (AcOH), 0.1 m NBu_4_PF_6_, 1 atm O_2_, 40 mL DMF. Working electrode: reticulated vitreous carbon. Potential: −760 mV vs Fc^+/0^.

The introduction of exogenous EPTMs can also increase the ORR activity. Stahl et al. accelerated the ORR using Co‐1 and BQ (EPTM‐10, **Figure** **15**). Controlled‐potential electrolysis (CPE) data showed that the current density of the Co‐1 + H_2_Q (EPTM‐9) system (0.54 mA cm^−2^) was double that of the Co‐1 system (0.27 mA cm^−2^).^[^ ^129^ ^]^ The ORR selectivity changed from 100% H_2_O_2_ to 60% H_2_O upon the use of EPTM‐9 as the exogenous EPTM with Co‐1, a mononuclear Co(sal)‐type catalyst (catalysts of this type generally mediate the ORR via the 2H^+^/2e^−^ pathway).^[^ ^114^ ^]^ The same group also demonstrated that under electrochemical ORR conditions, H_2_Q (2 equiv.) reacts with the Co(sal)–O_2_ adduct via hydrogen‐atom transfer (HAT) followed by PCET,^[^ ^130^ ^]^ which leads to the formation of H_2_O based on the rapid reduction of the formed H_2_O_2_ via an energetically favorable pathway (i.e., O_2_ → H_2_O_2_ → H_2_O).

#### 4.2.2 RMs in the Molecular Catalyst–Promoted ORR

**Figure 16:**
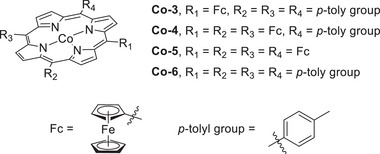
Co(TPP) complexes, **Co‐*n*** (***n*** = **3**–**6**), used to promote the electrochemical ORR.^[^ ^131^ ^]^

Kadish et al. (2014) prepared Co(TPP) complexes with different numbers of Fc groups at the *meso* positions of TPP and examined their abilities to promote the electrochemical ORR (**Figure** **16**, **Co‐3**–**6**).^[^ ^131^ ^]^ The redox events of these complexes were examined using UV–vis spectroelectrochemistry, and the covalently linked Fc groups were found to contribute to the ligand‐centered redox event. However, the authors did not determine whether these redox events are involved in the TLS or probe the impact of Fc substituents on the catalytic efficiency. Electrochemical studies revealed that the incorporation of the Fc substituent onto TPP caused an anodic shift in the reduction potential of Co(III)/(II), *E*_pc_. A selectivity study revealed that **Co‐3**–**5** exclusively afforded H_2_O_2_, whereas **Co‐6** afforded 40% H_2_O (**Table** **7**). The steric hindrance induced by the Fc groups prevented the formation of dimeric Co species, thereby accounting for the observed selectivity.

**Table 7:** Performances of **Co‐*n*** (***n*** = **3**–**6**) systems in the electrochemical ORR.^[^ ^131^ ^]^

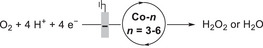				
Catalyst	*E*_pc_^[a]^ without O_2_ (V vs (SCE)	*E*_pc_ with O_2_ (V vs SCE)	*n*	H_2_O_2_ [%]
**Co‐3**	0.20	0.19	2.1	95
**Co‐4**	0.14	0.13	2.0	100
**Co‐5**	0.30	0.29	2.1	95
**Co‐6**	0.07	0.05	2.8	60

Reduction peak of Co(III)/Co(II). Experiments were performed in 1.0 M HClO_4_ saturated with N_2_ or air using an edge‐plane pyrolytic graphite disk electrode. The porphyrin catalyst was adsorbed on the electrode surface. SCE: saturated calomel electrode.

#### 4.2.3 PRs in the Molecular Catalyst–Promoted ORR

**Figure 17:**
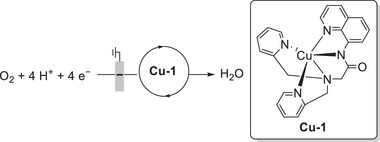
Electrochemical ORR catalyzed by Cu‐1.^[^ ^132^ ^]^

Biswas et al. (2020) synthesized a Cu(II) complex bearing a monoanionic pentadentate amidate ligand (dpaq) for use as an ORR electrocatalyst (**Figure** **17**, **Cu‐1**).^[^ ^132^ ^]^ When protonated, the carboxamido group of dpaq functioned as a PR, whereas the quinoline‐based moiety contributed the redox equivalent required for the conversion of **Cu‐1** and O_2_ into a (dpaqH)Cu(II)‐hydroperoxo species via internal 2H^+^/2e^−^ transfer. The number of electrons transferred (*n*_c_) in the ORR was calculated as 3.8 by rotating ring disk voltammetry, and chronoamperometric measurements revealed that the ORR proceeded via a stepwise (2e^−^ + 2e^−^) reduction pathway.

**Figure 18:**
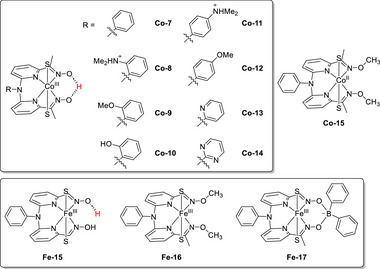
Molecular structures of bispyridine–dioxime complexes (**Co‐*n*** (***n*** = **7**–**15**) and **Fe‐*n*** (***n*** = **16**–**18**)) used as ORR electrocatalysts. S: MeCN.^[^ ^133^, ^134^, ^135^ ^]^

**Figure 19:**
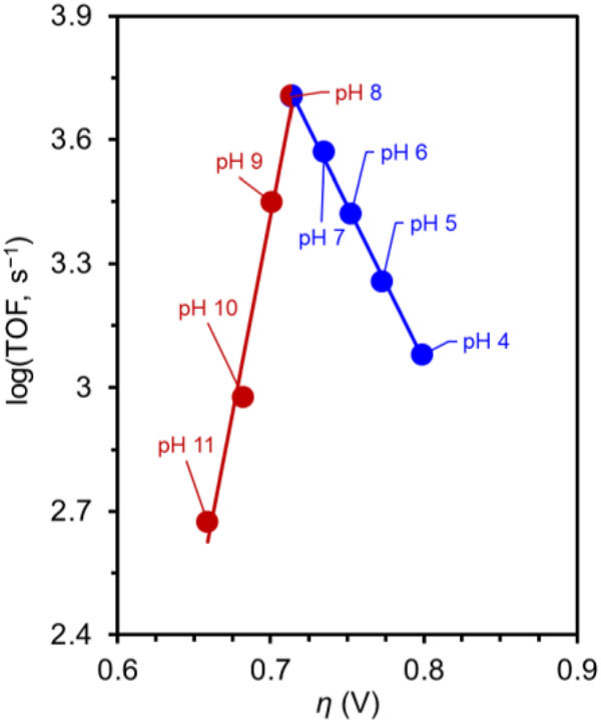
Results of the LFER analysis of the ORR catalyzed by **Co‐7** at different pH. General reaction conditions: [catalyst] = 2 mM, [CF_3_CO_2_H] = [CF_3_COO^−^] = 40 mM, O_2_‐saturated MeCN solution; [NBu_4_PF_6_] = 0.1 M; *v* = 25 mV s^−1^.^[^ ^133^ ^]^

Paria et al. prepared Co and Fe complexes featuring metal‐bound bispyridine–dioxime ligands and evaluated their performance as ORR electrocatalysts (**Figure** **18**).^[^ ^133^, ^134^, ^135^ ^]^ **Co‐7** selectively afforded H_2_O in an acidic buffer or acetonitrile (MeCN) solution, and the selectivity was strongly influenced by the ligand design and reaction medium.^[^ ^133^ ^]^ In 0.1 M phosphate buffer, the 4H^+^/4e^−^ pathway became unfavorable with increasing pH. Conversely, **Co‐7** retained the selectivity for this pathway under acidic and neutral conditions in 0.1 m acetate buffer (**Table** **8**). LFER analysis revealed that the highest reactivity was achieved at pH 8, with the corresponding TOF reaching ∼5000 s^−1^ at *η* = 0.7 V (**Figure** **19**). Experimental evidence indicated that the oxime unit in the SCS could act as a PR to deliver a proton. Computational findings suggested that the acetate ion mediated the transfer of a proton from the oxime backbone to the distal oxygen of the Co(III)−OOH species. Consequently, H_2_O was produced in near‐quantitative yields.

**Table 8:** Performances of **Co‐7** in the electrochemical ORR at different pH.^[^ ^133^ ^]^

pH	% H_2_O_2_	*η* [V]^a)^	TOF [s^−1^]
4	3	0.799	1.2 (±0.01) × 10^3^
5	4	0.773	1.8 (±0.01) × 10^3^
6	11	0.752	2.63 (±0.02) × 10^3^
7	25	0.735	3.72 (±0.02) × 10^3^
8	31	0.714	5.07 (±0.02) × 10^3^
9	42	0.701	2.81 (±0.01) × 10^3^
10	57	0.682	9.50 (±0.01) × 10^2^
11	65	0.659	4.71 (±0.01) × 10^2^

^a)^ *E*_1/2_ was considered to equal *E*_CAT_. General reaction conditions: [catalyst] = 2 mM, [CF_3_CO_2_H] = [CF_3_COO^−^] = 40 mM, O_2_‐saturated MeCN solution; [NBu_4_PF_6_] = 0.1 M; *v* = 25 mV s^−1^.

**Figure 20:**
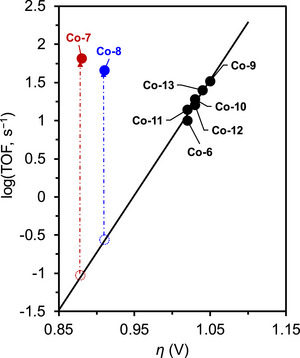
Results of the LFER analysis of the ORR catalyzed by **Co‐*n* (*n*** = **7**–**14**). General reaction conditions: [catalyst] = 2 mM, [CF_3_CO_2_H] = [CF_3_COO^−^] = 40 mM, O_2_‐saturated MeCN solution; [NBu_4_PF_6_] = 0.1 M; *ν* = 25 mV s^−1^. Reproduced with permission.^[^ ^135^ ^]^ Copyright 2025, American Chemical Society.

The same group modified the SCSs of bispyridine–dioxime Co complexes to improve the ORR activity (Figure 18, **Co‐7**–**15**).^[^ ^135^ ^]^ The trade‐off between the TOF and *η* was circumvented by installing *o*‐NHMe_2_^+^–C_6_H_4_ and *o*‐OMe–C_6_H_4_ substituents on the bispyridine–dioxime ligand (Figure 18, **Co‐8** and **Co‐9**). The TOFs of **Co‐8** and **Co‐9** were >1000 and 250 times greater, respectively, than those predicted based on the LFER between log(TOF) and *η* in a 1:1 CF_3_CO_2_H/CF_3_CO_2_^−^ buffer (**Table** **9** and **Figure** **20**). This outcome suggested that the TLS corresponded to protonation of the Co^III^(O_2_^•^) adduct, further indicating that the backbone *o*‐NHMe_2_^+^–C_6_H_4_ and *o*‐OMe–C_6_H_4_ substituents functioned as PRs and facilitated intramolecular PT to the Co^III^(O_2_^•^) adduct. Control experiments revealed that the oxime backbone of the bispyridine–dioxime ligand played a vital role as a proton‐exchange site.^[^ ^134^, ^135^ ^]^

**Table 9:** Performances of **Co‐*n*** (***n*** = **7**–**14**) systems in the electrochemical ORR.^[^ ^135^ ^]^

Catalyst	*E*_cat/2_ (V vs Fc^+/0^)^a)^	*η* [V]	TOF [s^−1^]
**Co‐7**	−0.51	1.02	10
**Co‐8**	−0.37	0.88	65
**Co‐9**	−0.40	0.91	46
**Co‐10**	−0.54	1.05	33
**Co‐11**	−0.52	1.03	19
**Co‐12**	−0.51	1.02	14
**Co‐13**	−0.50	1.03	16
**Co‐14**	−0.53	1.04	25

^a)^ *E*_CAT_ = *E*_cat/2_. General reaction conditions: [catalyst] = 2 mM, [CF_3_CO_2_H] = [CF_3_COO^−^] = 40 mM, O_2_‐saturated MeCN solution; [NBu_4_PF_6_] = 0.1 M; *ν* = 25 mV s^−1^.

Analogous Co and Fe complexes lacking an oxime unit did not catalyze the electrochemical ORR (Figure 18, **Co‐15, Fe‐16**, and **Fe‐17**). **Fe‐17** mediated the chemical ORR in the presence of Cp*_2_Fe as a reductant; however, the corresponding *k*_cat_ was 300 times lower than that of the oxime unit–containing **Fe‐15** (2.47 × 10^2^ vs 6.07 × 10^4^ M^−1^ s^−1^). Overall, these studies provided useful insights into the effects of the appended PRs and reaction media on the performance of mononuclear Co and Fe complexes as ORR (electro)catalysts.

**Figure 21:**
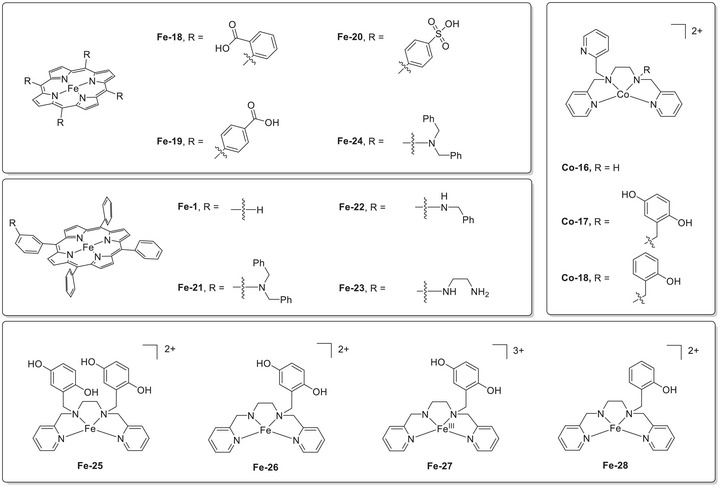
Structures of **Fe‐*n*** (***n*** = **1**, **18**–**28**) and **Co‐*n*** (***n*** = **16**–**18**) catalysts for the electrochemical ORR.^[^ ^136^, ^137^, ^138^, ^139^, ^140^, ^141^ ^]^

Mayer et al. (2012) evaluated the impact of carboxylate positioning on the kinetics and selectivity of the ORR catalyzed by Fe(TPP)‐based catalysts (**Figure** **21**, **Fe‐18** and **Fe‐19**).^[^ ^136^ ^]^ **Fe‐18** achieved a TOF of 200 s^−1^ and demonstrated a higher selectivity than **Fe‐19** (2% vs 9%). These results show that the PR effect observed when the carboxylate group was located at the *ortho* positions of the phenyl rings was superior to that observed when these groups were located at the *para* positions. Although the same group later revealed that the higher ORR rate of **Fe‐18** was due to its *η* exceeding that of **Fe‐19** under the same conditions, the role of PR in Fe(TPP)‐type catalysts for the ORR is worth exploring.^[^ ^137^ ^]^ The same series of Fe(TPP) catalysts with different substituents was prepared in the same study to examine the log(TOF)−*η* correlation for the ORR (e.g., **Fe‐20**). The identities of the catalyst and reaction media were found to be critical for optimizing catalyst performance, as the value of *η* is associated with the *E*_CAT_ and *E*_rxn_ (Equation 10). For instance, **Fe‐20** could achieve a higher ORR rate than **Fe‐18** at a lower *η*.

Dey et al. (2018) reported Fe(TPP)‐based ORR electrocatalysts with different amine substituents as pendant bases on the phenyl ring (Figure 21, **Fe‐21**–**23**).^[^ ^138^ ^]^ This catalyst design helped overcome the trade‐off relationship between log(TOF) and *η*, which was explained from both thermodynamic and kinetic perspectives. **Fe‐21**–**23** achieved ∼100‐fold higher TOFs than that of **Fe‐1** at similar *η* values in DMF solutions (**Table** **10**). Thermodynamically, the intramolecular hydrogen bonding between these pendant bases and the bound hydroperoxide stabilized the reaction intermediates; kinetically, amine protonation facilitated O–O bond cleavage in the bound hydroperoxide, which also increased the selectivity for H_2_O production via the 4H^+^/4e^−^ pathway.

**Table 10:** Performances of **Fe‐*n*** (***n*** = **1**, **18**–**28**) and **Co‐*n*** (***n*** = **16**–**18**) in the electrochemical ORR.^[^ ^136^, ^137^, ^138^, ^139^, ^140^, ^141^ ^]^

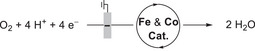					
Catalyst	TOF [s^−1^]	*η* [V]^h)^	Solvent	% H_2_O_2_	Refs.
**Fe‐1** ^a)^	27	1.08^i)^	DMF	−	[137]
**Fe‐1** ^b)^	1.59	1.20^i)^	MeCN	−	[139]
**Fe‐18** ^c)^	200	−	DMF	<2%	[136]
**Fe‐18** ^a)^	2000	1.18^i)^	DMF	−	[137]
**Fe‐19** ^a)^	15	1.04^i)^	DMF	〜9%	[136, 137]
**Fe‐20** ^a)^	1800	1.09^i)^	DMF	−	[137]
**Fe‐21** ^b)^	1601	1.095^i)^	DMF	−	[138]
**Fe‐21** ^d)^	10.30	1.08^i)^	MeCN	−	[139]
**Fe‐22** ^b)^	3960	1.052^i)^	DMF	−	[138]
**Fe‐23** ^b)^	2970	1.08^i)^	DMF	−	[138]
**Fe‐24** ^d)^	12.30	1.01^i)^	MeCN	−	[139]
**Fe‐25** ^e)^	14.6	0.58^j)^	MeCN	92	[141]
**Fe‐26** ^f)^	3.3	0.42^j)^	MeCN	83	[141]
**Fe‐27** ^f)^	3.2	0.37^j)^	MeCN	81	[141]
**Fe‐28**	−	0.41^j)^	MeCN	26	[141]
**Co‐16**	−	−	MeCN	−	[140]
**Co‐17** ^g)^	0.31	0.33^j)^	MeCN	61	^[^ ^140^ ^]^ ^[^ ^141^ ^]^
**Co‐18** ^g)^	0.32	0.31^j)^	MeCN	11	[140, 141]

^a)^ 20 mM [DMF‐H][OTf] under 1 atm O_2_;

^b)^ 50 mM [DMF‐H][OTf] at 1 atm O_2_;

^c)^ 20 mM [DMF‐H][OTf] under 1 atm O_2_;

^d)^ 5 mM TsOH at 1 atm O_2_;

^e)^ 0.15 mM catalyst, 400 mM acetate buffer with 6.3 mM O_2_;

^f)^ 0.1 mM catalyst and 400 mM acetate buffer with 6.3 mM O_2_;

^g)^ 1.0 mM catalyst and 100 mM acetate buffer (AcOH: NaOAc = 1:1) with 6.3 mM O_2_;

^h)^ Calculated using Equation (10);

^i)^ *E*_CAT_ = *E*_1/2_;

^j)^ *E*_CAT_ = *E*_cat/2_.

**Figure 22:**
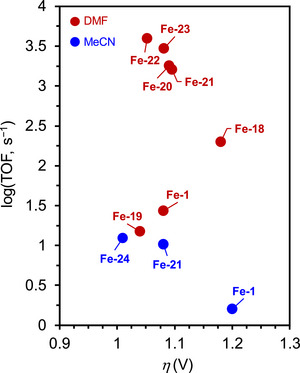
Results of the LFER analysis of **Fe‐*n*** (***n*** = **1**, **18**–**24**) obtained for the electrochemical ORR performed in DMF (red) and MeCN (blue).^[^ ^136^, ^137^, ^139^ ^]^

In addition to **Fe‐21**, a subsequent study reported **Fe‐24**, which features four *N*,*N*‐dibenzylamino substituents at the *ortho* positions of the phenyl rings (Figure 21).^[^ ^139^ ^]^ The catalytic efficiencies of **Fe‐1**, **Fe‐21,** and **Fe‐24** were evaluated in MeCN based on log(TOF)−*η* relationships (**Figure** **22**). This LFER analysis showed that **Fe‐21** and **Fe‐24** exhibited TOFs that markedly exceeded the predicted values and that of **Fe‐1**, confirming that the *N*,*N*‐dibenzylamino substituents were capable of mediating heterolytic O−O bond cleavage in the Fe^III^−OOH species. With respect to thermodynamic advantages, **Fe‐21** and **Fe‐24** catalyzed the ORR at *η* values ∼100 mV lower than that of **Fe‐1**. These results highlight that the trade‐off between the reaction rate and driving force can be avoided by utilizing PRs in the ORR.

**Figure 23:**
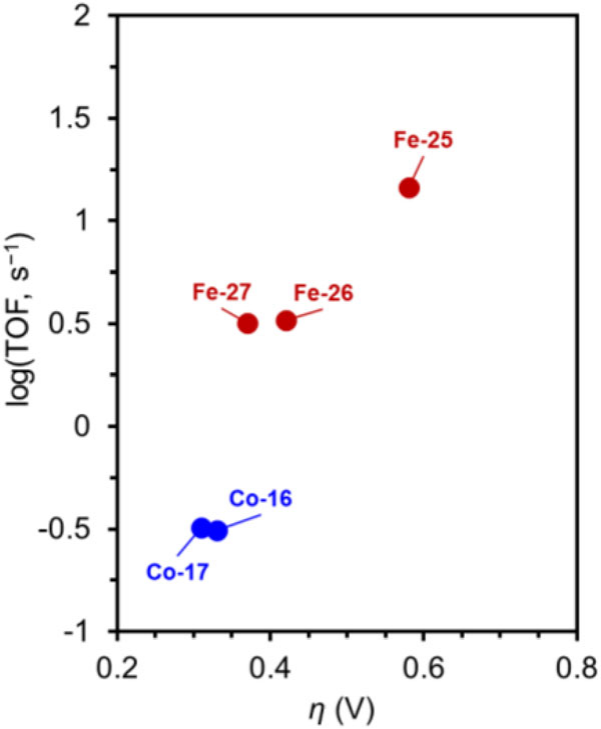
Results of the LFER analysis of **Fe‐*n*** (***n*** = **25**–**28**) and **Co‐*n*** (***n*** = **17** and **18**) obtained for the electrochemical ORR.^[^ ^141^ ^]^

In 2022, Goldsmith et al. developed Co^II^ complexes with polydentate N‐donor ligands (Figure 21, **Co‐16**–**18**).^[^ ^133^, ^140^, ^141^ ^]^ Unlike the inactive **Co‐16**, **Co‐17** and **Co‐18** were electroactive in the ORR and exhibited similar TOFs (Table 10 and Figure 21). This observation indicates that the phenolic components of **Co‐17** and **Co‐18** facilitated intramolecular PT, thereby enhancing the ORR kinetics. **Co‐17**, featuring a dangling H_2_Q group, exhibited a higher selectivity for H_2_O than **Co‐18**, which contained an appended phenol group (Table 10, *n*_c_: 3.5 vs 2.2). This finding indicates that the H_2_Q moiety of **Co‐16** acted as a 2H^+^/2e^−^ reservoir and influenced the formation of the [Co^III^−OH]^2+^ intermediate, which is proposed to be the product‐determining step. The same group synthesized Fe^II^ and Fe^III^ complexes featuring ligand scaffolds similar to H_2_Q or phenol groups and evaluated their ORR performances (Figure 21, **Fe‐25**–**28**).^[^ ^141^ ^]^ **Fe‐25**, containing two H_2_Q groups, exhibited a TOF (14.6 s^−1^) several times higher than those of the other Fe catalysts (Table 10 and **Figure** **23**). Additionally, **Fe‐25** favored H_2_O over H_2_O_2_ due to the non‐innocence of the covalently linked H_2_Q. This effect was similar to that observed for analogous Co complexes.^[^ ^133^, ^140^ ^]^

**Figure 24:**
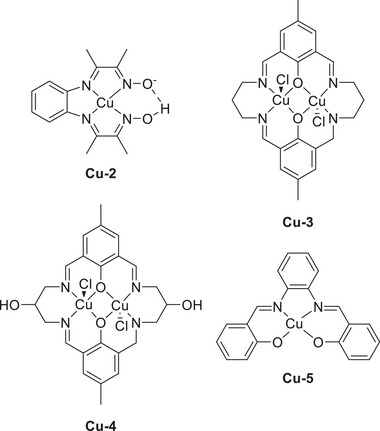
Structures of **Cu‐*n*** (***n*** = **2**–**5**) catalysts for the electrochemical ORR and water oxidation.^[^ ^142^, ^143^ ^]^

**Figure 25:**
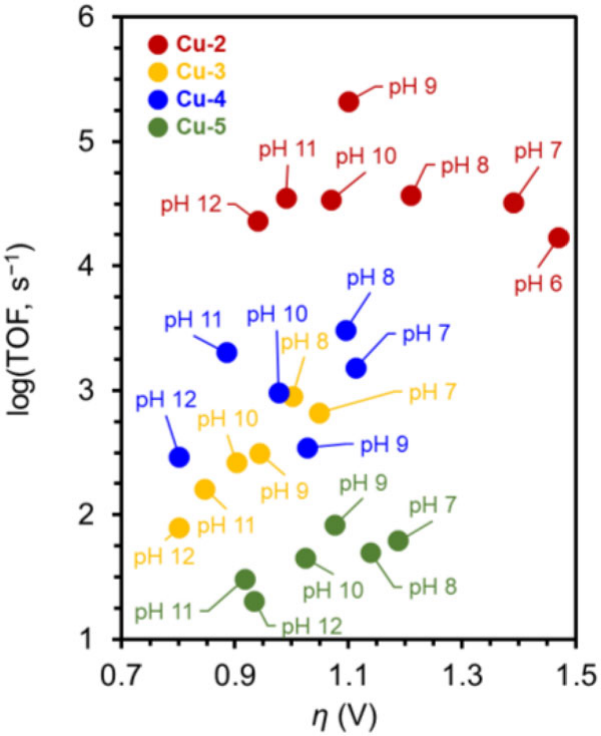
Results of the LFER analysis of **Cu‐*n*** (***n*** = **2**–**5**) catalysts used in the electrochemical ORR.^[^ ^142^, ^143^ ^]^

Ghosh et al. prepared a tetradentate mononuclear Cu complex (**Figure** **24**, **Cu‐2**) and examined its ability to promote the electrochemical ORR and WOR (see Section 4.3 for details).^[^ ^142^ ^]^ The diimine–dioxime framework provided a labile coordination geometry and served as a PR to enhance the stability of the reduced Cu species. FOWA revealed that **Cu‐2** electrochemically catalyzed the ORR or WOR through a bimolecular pathway and generated a *µ*‐1,2‐peroxo‐dicopper intermediate ([Cu^II^−O_2_−Cu^I^]^−^). Computational studies implied that a mononuclear ([Cu^III^−OH]^+^) was probably formed upon the O−O bond cleavage of [Cu^II^−O_2_−Cu^I^]^−^, which resulted in four‐electron O_2_ reduction. The selective 4H^+^/4e^–^ reduction of O_2_ to H_2_O in the presence of **Cu‐2** was observed over an extensive pH range (6.0–12.0), with the highest catalytic efficiency (TOF = 2.1 × 10^5^ s^−1^ at *η* = 1.10 V) recorded at pH 9.0 (**Table** **11** and **Figure** **25**).

**Table 11:** Performances of **Cu‐*n*** (***n*** = **2**–**5**) in the electrochemical ORR.^[^ ^142^, ^143^ ^]^ ^a)^

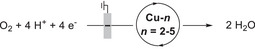				
Catalyst	pH	TOF [s^−1^]	*η* [V]^b)^	Refs.
**Cu‐2**	6	1.7 × 10^4^	1.47	[142]
**Cu‐2**	7	3.2 × 10^4^	1.39	[142]
**Cu‐2**	8	3.7 × 10^4^	1.21	[142]
**Cu‐2**	9	2.1 × 10^5^	1.10	[142]
**Cu‐2**	10	3.4 × 10^4^	1.07	[142]
**Cu‐2**	11	3.5 × 10^4^	0.99	[142]
**Cu‐2**	12	2.3 × 10^4^	0.94	[142]
**Cu‐3**	7	664.589	1.048	[143]
**Cu‐3**	8	899.618	1.001	[143]
**Cu‐3**	9	313.223	0.943	[143]
**Cu‐3**	10	260.894	0.903	[143]
**Cu‐3**	11	161.623	0.847	[143]
**Cu‐3**	12	78.669	0.802	[143]
**Cu‐4**	7	1521.027	1.113	[143]
**Cu‐4**	8	3066.521	1.095	[143]
**Cu‐4**	9	346.724	1.028	[143]
**Cu‐4**	10	950.114	0.977	[143]
**Cu‐4**	11	2011.736	0.885	[143]
**Cu‐4**	12	290.256	0.802	[143]
**Cu‐5**	7	61.743	1.188	[143]
**Cu‐5**	8	50.072	1.138	[143]
**Cu‐5**	9	83.183	1.076	[143]
**Cu‐5**	10	44.813	1.025	[143]
**Cu‐5**	11	30.36	0.917	[143]
**Cu‐5**	12	20.254	0.934	[143]

^a)^ The TOF values were obtained using CV measurements in a buffered aqueous solution containing 0.1 m Na_2_SO_4_. *v* = 1.0 V s^−1^, 25 °C;

^b)^ Calculated using Equation (10) and *E*_CAT_ = *E*_cat/2_.

Two other dinuclear Cu complexes bearing tetradentate N_2_O_2_‐type ligands (Figure 24, **Cu‐3** and **Cu‐4**) also showed bidirectional reactivities in the ORR and WOR.^[^ ^143^ ^]^ The two peripheral −OH groups on the macrocyclic ligand of **Cu‐4** were suggested to function as PRs during catalysis. Therefore, **Cu‐4** exhibited a higher catalytic efficiency than **Cu‐3** and the control mononuclear Cu(sal) catalyst (**Cu‐5**), as shown in Table 11 and Figure 23. The ORR study was conducted in aqueous solutions at pH 7–12, and both **Cu‐3** and **Cu‐4** exhibited the highest TOF at pH 8 (**Cu‐3**: ∼1000 s^−1^, **Cu‐4**: ∼3000 s^−1^). No clear correlation between log(TOF) and *η* for **Cu‐3**–**5** was observed, possibly because pH affected the catalytic pathway (Figure 25).

### 4.3 Water Oxidation Reaction (WOR)

#### 4.3.1 RMs in the Molecular Catalyst–Promoted WOR

**Figure 26:**
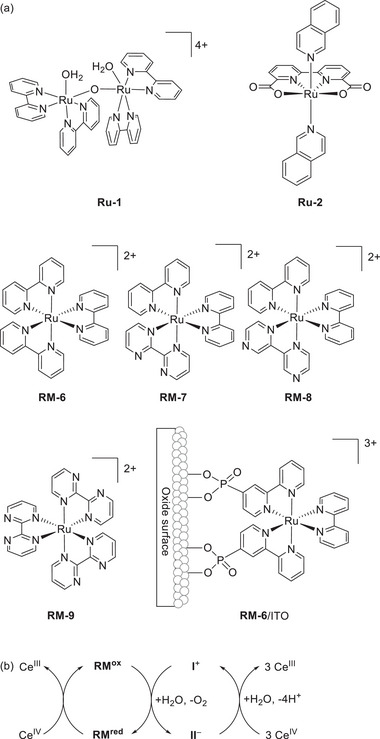
(a) Structures of **Ru‐*n*** (***n*** = **1** and **2**)**/RM‐*n*** (***n*** = **6**–**9**) systems used to promote the electrochemical WOR.^[^ ^146^, ^147^ ^]^ (b) Proposed catalytic cycle for the chemical WOR promoted by the blue dimer in the presence of **RM‐6**–**9**.^[^ ^146^, ^147^ ^]^

**Figure 27:**
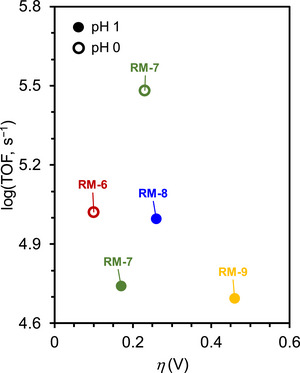
Results of the LFER analysis of **Ru‐1/RM‐*n*** (***n*** = **6**–**9**) systems used to promote the chemical WOR.^[^ ^146^ ^]^

Meyer et al. (1982) reported a dinuclear *µ*‐oxo‐bridged Ru complex, *cis*,*cis*‐[(bpy)_2_(H_2_O)Ru(*µ*‐O)Ru(H_2_O)(bpy)_2_]^4+^ (**Figure** **26**a, **Ru‐1**) as the first molecular water oxidation catalyst (WOC). This catalyst is also known as the “blue dimer” because of the blue color of its aqueous solutions.^[^ ^144^, ^145^ ^]^ At pH 4, **Ru‐1** achieved a TON of 13.2 and TOF of 0.0042 s^−1^ based on O_2_ evolution at *η* = 0.262 V in the presence of ceric ammonium nitrate (CAN) as a sacrificial oxidant.^[^ ^15^, ^145^ ^]^ In 2008, the same group investigated the mechanism of the (electro)chemical WOR catalyzed by **Ru‐1** in the presence of various Ru mediators (Figure 26a, **RM‐6**–**9**).^[^ ^146^ ^]^ The second‐order rate constants for CAN consumption by catalytically active dimeric intermediates (**I**: [(bpy)_2_(HO_2_)Ru^III^ORu^V^(O)(bpy)_2_]^3+^, **II**: [(bpy)_2_(HO_2_)Ru^IV^ORu^IV^(OH)(bpy)_2_]^4+^) increased 10−30‐fold in the presence of **RM‐6**–**9** (**Table** **12**, *k* **_I_** and *k* **_II_**), which was attributed to the mediated oxidation of the peroxidic species **I** and **II**. These findings suggest that the chemical WOR can be optimized by accelerating inner‐sphere ET using exogenous RMs (Figure 26b). **Ru‐1**/**RM‐7** demonstrated the highest catalytic activity among the other RMs, with *η* equaling only 230 mV (**Figure** **27**).

**Table 12:** Performances of **Ru‐*n*** (***n*** = **1** and **2**)**/RM‐*n*** (***n*** = **6**–**9**) systems in the chemical WOR.^[^ ^146^ ^]^

Catalyst	Mediator	pH	*k*_I_ [M^−1^ s^−1^]^a)^	*k*_II_ [M^−1^ s^−1^]^b)^	TOF [s^−1^]	*η* [V]	Refs.
**Ru‐1**	–	0	80	–	4.4 × 10^3^ ^c)^	–	[146]
**Ru‐1**	–	1	–	180	9.9 × 10^3^ ^c)^	–	[146]
**Ru‐1**	**RM‐6**	1	–	1900	1.05 × 10^5^ ^c)^	0.10^e)^	[146]
**Ru‐1**	**RM‐7**	0	1000	–	5.5 × 10^4^ ^c)^	0.23^e)^	[146]
**Ru‐1**	**RM‐7**	1	–	5500	3.03 × 10^5^ ^c)^	0.23^e)^	[146]
**Ru‐1**	**RM‐8**	0	1800	–	9.9 × 10^4^ ^c)^	0.26^e)^	[146]
**Ru‐1**	**RM‐9**	0	900	–	4.95 × 10^4^ ^c)^	0.46^e)^	[146]
**Ru‐2**	–	7.2	–	–	0.194^d)^	0.295^f)^	[147]
**Ru‐2**	**RM‐6**	7.2	–	–	5.25^d)^	0.02^f)^	[147]

^a)^ pH 0: 1.0 M HNO_3_;

^b)^ pH: 1: 0.1 M HNO_3_;

^c)^ Calculated from *k* **_I_** or *k* **_II_**;

^d)^ Calculated using Equation (6);

^e)^ *E*_CAT_ = *E*_onset_.

^f)^ *E*_CAT_ = *E*_CPE_.

In a subsequent study, Meyer et al. achieved a remarkable rate enhancement using **Ru‐2** (Figure 26a) and an indium‐doped tin oxide (ITO) electrode with an immobilized Ru mediator (Figure 26a, **RM‐6**).^[^ ^147^ ^]^ At pH 7.2, the ratio of the catalytic current to the non‐catalytic current (*i*_c_/*i*_p_) for **Ru‐2/RM‐6** was approximately five times higher than that for **Ru‐2**, implying that the immobilized **RM‐6** promoted heterogeneous ET between **Ru‐2** and the electrode and thereby selectively catalyzed the conversion of H_2_O to O_2_. Additionally, **RM‐6** achieved a 275 mV decrease in *η*, which was calculated from the potential required to reach an *i*_c_/*i*_p_ ratio of 1 at 1.4 V vs SCE in the absence of **RM‐6**.

**Figure 28:**
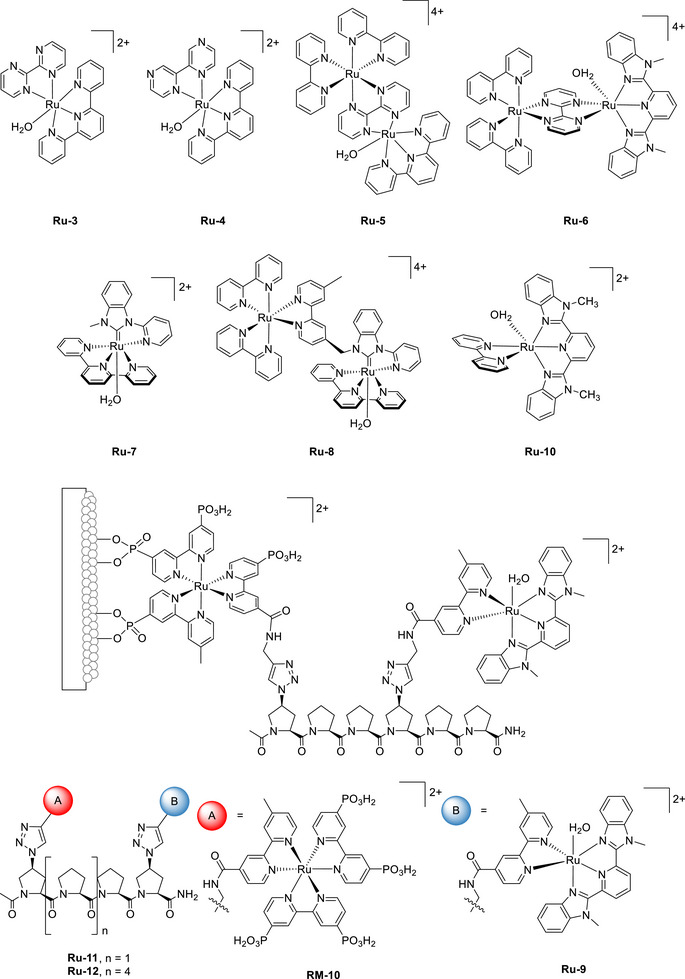
Structures of **Ru‐*n*** (***n*** = **3**–**12**) and **RM‐10** systems used to promote the electrochemical WOR.^[^ ^144^, ^145^, ^146^, ^147^, ^148^, ^149^, ^150^, ^151^, ^152^, ^153^, ^154^, ^155^ ^]^

In addition to exogenous RMs, endogenous RMs, i.e., those covalently connected to a molecular catalyst, can also be used to boost the activity of molecular catalysts. Meyer et al. (2009) examined **Ru‐3**–**6,** among which **Ru‐5** and **Ru‐6** are assemblies of **Ru‐3** with tpy and 2,6‐bis(1‐methyl‐benzimidazol‐2‐yl)pyridine (Mebimpy), respectively (**Figure** **28**).^[^ ^148^ ^]^ When CAN was used as a sacrificial oxidant in 0.1 M HNO_3_, the TOF of **Ru‐3** was recorded as 7.5 × 10^−4^ s^−1^ at *η* = 0.479 V.^[^ ^149^ ^]^ Compared with **Ru‐3**, **Ru‐5**, and **Ru‐6**, which were anchored on the ITO electrode via phosphonate linkers, these showed remarkably higher TONs of 8900 and 28000 and TOFs of 0.3 and 0.6 s−1, respectively, highlighting the crucial role of the assembled mediator in WOCs.

The same group prepared **Ru‐7** and **Ru‐8** (Figure 28). **Ru‐8** was designed by integrating **Ru‐7** and a Ru(bpy)_3_ derivative as the RM to investigate the chemical WOR.^[^ ^150^ ^]^ Mechanistic investigations revealed that **Ru‐8** exhibited a distinct reaction pathway compared to **Ru‐7**. A kinetic study of O_2_ evolution showed that the reactivity of **Ru‐8** was eight times greater than that of **Ru‐7**. Meyer et al. (2014) developed another dyad assembly, namely a Ru‐based WOC in which **Ru‐9** was covalently attached to a fluorine‐doped tin oxide electrode–immobilized Ru chromophore (**RM‐10**) through oligoproline peptides (Figure 28, **Ru‐11** and **Ru‐12**).^[^ ^151^ ^]^ The TOFs of **Ru‐11** and **Ru‐12** were determined by CV as 0.85 and 0.57 s^−1^, respectively (**Table** **13**). Thus, **Ru‐11** and **Ru‐12** were more efficacious than [Ru(Mebimpy)(bpy)(H_2_O)]^2+^ (**Ru‐10**), which featured a TOF of 0.027 s^−1^ under identical conditions.

**Table 13:** Performances of **Ru‐*n*** (***n*** = **3**–**12**) in the electrochemical WOR.^[^ ^144^, ^145^, ^146^, ^147^, ^148^, ^149^, ^150^, ^151^, ^152^, ^153^, ^154^, ^155^ ^]^

Catalyst	pH	TOF [s^−1^]	*η* [V]	Refs.
**Ru‐3**	1	7.5 × 10^−4^ ^c)^	0.479^f)^	[149]
**Ru‐4**	1	1.4 × 10^−3^ ^c)^	>0.479^f)^	[149]
**Ru‐5**	0	0.3^a)^	0.57^f)^	[148]
**Ru‐6**	0	0.6^a)^	0.57^f)^	[148]
**Ru‐7**	1	1.2 × 10^−2^ ^a)^	0.529^e)^	[150]
**Ru‐8**	1	0.102^a)^	0.329^e)^	[150]
**Ru‐10**	1	0.027^b)^	0.57^d)^	[151]
**Ru‐11**	1	0.85^b)^	0.57^d)^	[151]
**Ru‐12**	1	0.57^b)^	0.57^d)^	[151]

^a)^ Calculated from *k*_obs_;

^b)^ Calculated using Equation (6);

^c)^ TOF = *k*_obs_;

^d)^ *E*_CAT_ = *E*_1/2_;

^e)^ *E*_CAT_ = *E*_onset_;

^f)^ *E*_CAT_ = *E*_CPE_.

**Figure 29:**
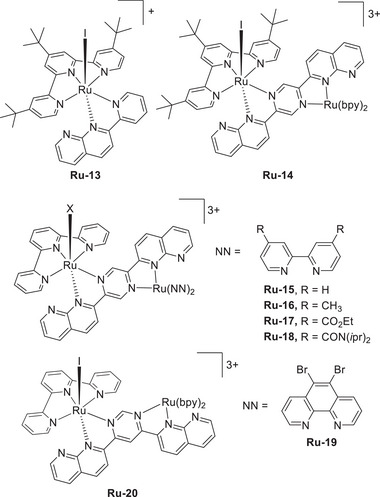
Structures of **Ru‐*n*** (***n*** = **13**–**20**) catalysts used in the electrochemical WOR.^[^ ^156^, ^157^ ^]^

RM‐modified catalysts can also promote the photochemical WOR, as demonstrated by Thummel et al., who synthesized **Ru‐13** and **Ru‐14** by connecting a Ru photosensitizer (PS) to a Ru‐centered catalytic moiety using a pyrazine‐derived linker (**Figure** **29**).^[^ ^156^ ^]^ Under blue‐light irradiation, **Ru‐14** achieved a TON of 134 (6 h) based on O_2_ liberation (**Table** **14**), a value markedly exceeding that of a mixture of **Ru‐13** and **RM‐6** under the same conditions (TON = 6). This result indicates that effective intramolecular ET can promote the WOR and can be achieved through rational catalyst design. Thummel et al. also developed hybrid dinuclear Ru complexes containing mononuclear Ru polypyridyl sensitizers (Figure 29, **Ru‐15**–**20**).^[^ ^157^ ^]^ The ET driving force increased with the increasing difference between the excited‐state oxidation potential of the sensitizer component (E*_1/2_^ox^) and the ground‐state oxidation potential of the catalyst (*E*_1/2_^ox^), resulting in different photocatalytic reactivities in the WOR.

**Table 14:** Performances of **Ru‐*n*** (***n*** = **13**–**20**) in the electrochemical WOR.^[^ ^156^, ^157^ ^]^

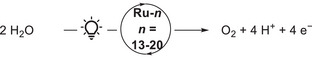				
Catalyst	TON	*E*_1/2_^ox^ (V vs SCE)	*E*_1/2_^red^ (V vs SCE)	Refs.
**Ru‐13** ^a)^	6^c)^	0.72	−1.12 −1.62^ir^	[156]
**Ru‐14** ^b)^	134^c)^	0.84 1.54^ir^	−0.56^ir^ −10^ir^	[156]
**Ru‐15**	35^d)^	0.93 1.52^ir^	−0.44 −0.80	[157]
**Ru‐16**	20^d)^	0.97 1.49^ir^	−0.41 −0.77	[157]
**Ru‐17**	47^d)^	0.94 1.66^ir^	−0.43 −0.77	[157]
**Ru‐18**	50^d)^	0.95 1.59^ir^	−0.43 −0.78	[157]
**Ru‐19**	68^d)^	0.94 1.64^ir^	−0.43 −0.76	[157]
**Ru‐20**	48^d)^	0.95 1.56^ir^	−0.45 −0.89	[157]

^a)^ 0.2 µmol cat. and 0.8 µmol [Ru(bpy)_3_]Cl_2_ in the presence of 0.04 mmol Na_2_S_2_O_8_ and blue light;

^b)^ 0.0063 µmol in the presence of 0.04 mmol Na_2_S_2_O_8_ and blue light;

^c)^ TON at 6 h;

^d)^ TON at 1 h. ir = irreversible voltammetric wave.

#### 4.3.2 PRs in the Molecular Catalyst–Promoted WOR

**Figure 30:**
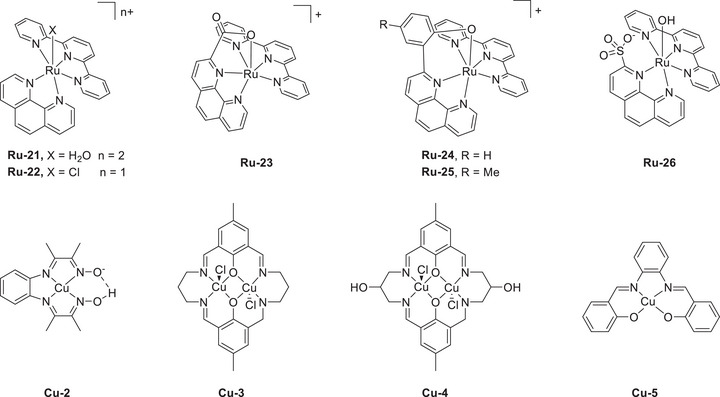
Structures of **Ru‐*n*** (***n*** = **21**–**26**) and **Cu‐n** (***n*** = **2**–**5**) catalysts used to promote the electrochemical WOR.^[^ ^142^, ^143^, ^158^, ^159^, ^160^ ^]^

Ru complexes with tpy‐ and phen‐based ligand motifs were reported as WOCs by the groups of Thummel and Grotjahn (**Figure** **30**, **Ru‐21**–**26**).^[^ ^158^, ^159^, ^160^ ^]^ In these complexes, the ─CO_2_ and ─SO_3_H moieties on phen functioned as PRs (**Ru‐23** and **Ru‐26**).^[^ ^158^, ^159^ ^]^ In the chemical WOR using CAN as the sacrificial oxidant, **Ru‐23** and **Ru‐26** achieved TONs of 700 (10 h) and 7400 (25 h), respectively. Although the reaction conditions were not identical, the origin of the large TON difference between **Ru‐23** and **Ru‐26** may potentially be due to the hemilability of the carboxylate and sulfonate groups, which deserves further investigation. Initial rate analysis revealed that **Ru‐26** featured a rate constant (0.88 s^−1^) six times higher than that of **Ru‐23** (0.15 s^−1^) under the same conditions. Additionally, Ru complexes bearing an identical ligand framework but lacking PRs exhibited lower TONs after 24 h (**Ru‐21**: 45, **Ru‐22**: 400).^[^ ^160^ ^]^ Thus, by facilitating PT, PRs can play a vital role in increasing the activities of molecular WOCs.

**Figure 31:**
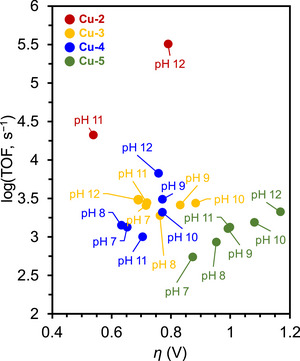
Results of the LFER analysis of **Cu‐*n*** (***n*** = **2**–**5**) catalysts used to promote the electrochemical WOR.^[^ ^142^, ^143^ ^]^

Dutta et al. (2021) developed a mononuclear Cu complex bearing a diimine–dioxime ligand (Figure 24, **Cu‐2**).^[^ ^142^ ^]^ This fluxional ligand features a hemilabile coordination geometry, with the peripheral hydroxyl group facilitating PT and stabilizing the oxidized/reduced Cu center. Consequently, **Cu‐2** exhibited an exceptional bidirectional activity for the electrochemical WOR under alkaline conditions (**Table** **15** and **Figure** **31**). The same group also reported dinuclear Cu complexes with and without peripheral hydroxyl groups (Figure 24, **Cu‐3** and **Cu‐4**).^[^ ^143^ ^]^ The comparison of **Cu‐3–5** performances for the WOR conducted at pH 7−12 (Table 15) revealed that the TOF of **Cu‐4** at *η* = 0.758 V reached 6738 s^−1^ at pH 12. Owing to the cooperative interaction between two Cu centers and the proximal hydroxyl group acting as a PR, **Cu‐4** exhibited a WOR performance exceeding those of the structurally analogous **Cu‐3** and **Cu‐5**.

**Table 15:** Performances of **Ru‐*n*** (***n*** = **21**–**26**) and **Cu‐*n*** (***n*** = **2**–**5**) in the electrochemical WOR.

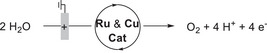					
Catalyst	pH	TOF [s^−1^]	TON	*η* [V]^i^ ^)^	Refs.
**Ru‐21** ^a)^	7	280	450^f^ ^)^	−	[160]
**Ru‐22** ^a)^	7	20	400^f^ ^)^	−	[160]
**Ru‐23** ^b)^	1	0.15	700^g^ ^)^	−	[158]
**Ru‐24** ^b)^	1	0.13	700^g^ ^)^	−	[158]
**Ru‐25** ^b)^	1	0.06	>300^g^ ^)^	−	[158]
**Ru‐26** ^c)^	7	2598	−	0.8	[159]
**Ru‐26** ^d)^	1.1	0.88	7400^h^ ^)^	−	[159]
**Cu‐2** ^e)^	11	2.1 × 10^4^	−	0.54	[142]
**Cu‐2** ^e)^	12	3.2 × 10^5^	−	0.79	[142]
**Cu‐3** ^e)^	7	2558	−	0.716	[143]
**Cu‐3** ^e)^	8	1882	−	0.762	[143]
**Cu‐3** ^e)^	9	2582	−	0.831	[143]
**Cu‐3** ^e)^	10	2724	−	0.882	[143]
**Cu‐3** ^e)^	11	2784	−	0.719	[143]
**Cu‐3** ^e)^	12	3045	−	0.689	[143]
**Cu‐4** ^e)^	7	1327	−	0.652	[143]
**Cu‐4** ^e)^	8	1408	−	0.632	[143]
**Cu‐4** ^e)^	9	3075	−	0.771	[143]
**Cu‐4** ^e)^	10	2100	−	0.771	[143]
**Cu‐4** ^e)^	11	1003	−	0.704	[143]
**Cu‐4** ^e)^	12	6738	−	0.758	[143]
**Cu‐5** ^e)^	7	550	−	0.873	[143]
**Cu‐5** ^e)^	8	860	−	0.952	[143]
**Cu‐5** ^e)^	9	1275	−	0.991	[143]
**Cu‐5** ^e)^	10	1544	−	1.080	[143]
**Cu‐5** ^e)^	11	1329	−	0.998	[143]
**Cu‐5** ^e)^	12	2115	−	1.168	[143]

^a)^ 0.4 mM catalyst, 0.2 M CAN;

^b)^ 20 µM catalyst, 0.2 M CAN;

^c)^ 0.125 mM catalyst in 0.0294 M phosphate buffer;

^d)^ 5 µM catalyst, 0.2 m CAN;

^e)^ 0.1 M Na_2_SO_4_ buffer solution;

^f)^ TON at 24 h;

^g)^ TON at 10 h;

^h)^ TON at 25 h;

^i)^ Calculated using Equation (10) and *E*_CAT_ = *E*_cat/2_.

**Figure 32:**
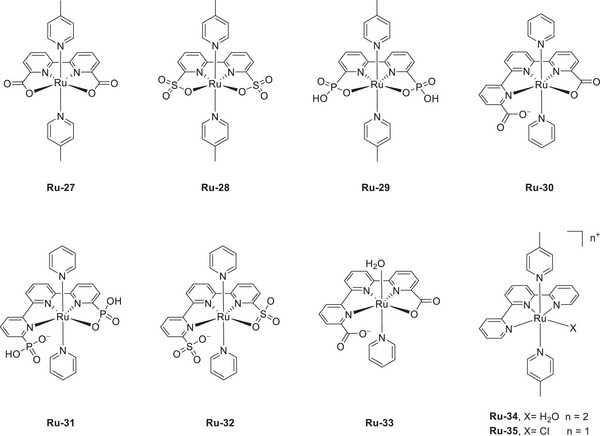
Structures of **Ru‐*n*** (***n*** = **27**–**35**) catalysts used to promote the electrochemical WOR.^[^ ^34^, ^160^, ^161^, ^162^, ^163^, ^164^, ^165^, ^166^, ^167^, ^168^ ^]^

**Figure 33:**
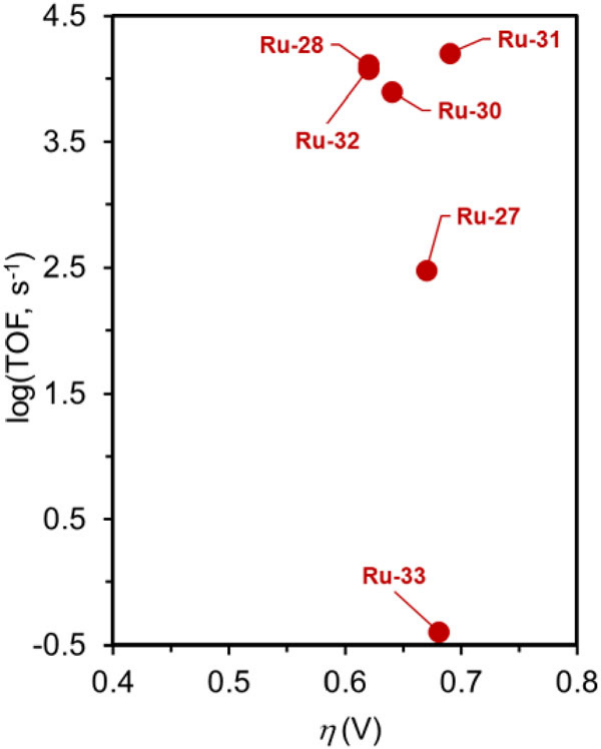
Results of the LFER analysis of **Ru‐*n*** (***n*** = **27**–**35**) catalysts used to promote the electrochemical WOR.^[^ ^162^, ^163^, ^164^, ^166^ ^]^

**Figure 34:**
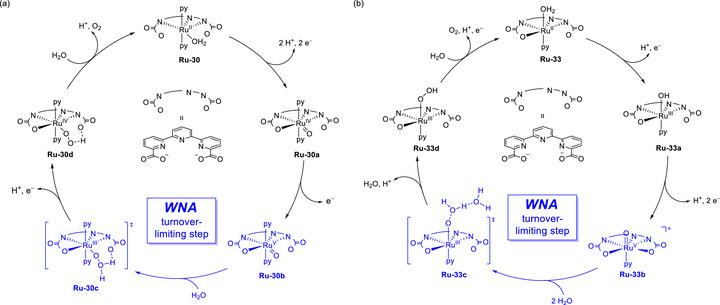
a) Catalytic cycle of **Ru‐30** with dangling carboxylate groups aligned in the same orientation as the incoming water molecule. b) Catalytic cycle of **Ru‐33** with dangling carboxylate groups oriented perpendicularly to the incoming water molecule. Reproduced with permission.^[^ ^50^ ^]^ Copyright 2023, Royal Society of Chemistry.

In 2019, Sun and Llobet summarized the importance of seven‐coordinated Ru complexes and reaction intermediates.^[^ ^34^, ^161^ ^]^ The WOR performances of representative seven‐coordinate Ru complexes bearing –CO_2_H, –SO_3_H, or –PO_3_H_2_ substituents incorporated into the PCS as pendant arms (**Figure** **32**, **Ru‐27**–**33**)^[^ ^162^, ^163^, ^164^, ^165^, ^166^ ^]^ were evaluated based on TOF and *η* (**Table** **16**) through log(TOF)−*η* analysis (**Figure** **33**). **Ru‐28** and **Ru‐31** at pH 7 exhibited the most outstanding activity, showing *η* values comparable with those of **Ru‐27** and **Ru‐30**–**32**. Moreover, **Ru‐30**–**32**, which incorporate PRs, exhibited notably higher TOFs than the structurally similar **Ru‐34** and **Ru‐35**, which lack PRs (Table 16). The effects of carboxylates, sulfonates, and phosphonates in the SCSs of Ru‐based WOCs were comprehensively discussed with regard to the WOR kinetics and thermodynamics in our previous publication.^[^ ^50^ ^]^ The orientation of these pendant bases with respect to the Ru^III^−OOH unit is a crucial factor determining their abilities to act as effective PRs (**Figure** **34**, **Ru‐30** vs **Ru‐33**). A mechanistic study revealed that the hemilabile coordination of carboxylate, sulfonate, and phosphonate moieties allows them to act as pendant bases and thereby facilitate the intramolecular PT of the Ru^III^−OOH intermediate to close the catalytic cycle.^[^ ^162^, ^167^ ^]^

**Table 16:** Performances of **Ru‐*n*** (***n*** = **27**–**35**) in the electrochemical WOR.

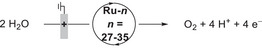							
Catalyst	pH	TOF [s^−1^]	TON	*E*_onset_ (V vs RHE)	*η* [V]	Mechanism	Refs.
**Ru‐27** ^a)^	1	41.2	2000	1.5	−	I2M	[161, 165]
**Ru‐27**	1^b)^ 7^c)^	7 300	−	1.55	0.67^g)^	I2M	[164, 166]
**Ru‐28**	1^b)^ 7^c)^	160 12 900	−	1.65	0.62^g)^	I2M	[164, 166]
**Ru‐30** ^d)^	7 8 10	8000 25 000 50 000	2.7 × 10^7^ ^e)^	1.70–1.80	0.64^g)^	WNA	[162]
**Ru‐31** ^d)^	7	16 000	−	1.76	0.69^g)^	WNA	[163]
**Ru‐32** ^d)^	7	12 000	−	1.76	0.62^g)^	WNA	[164]
**Ru‐33**	7	0.4	−	−	0.68	WNA	[167]
**Ru‐34** ^b)^	7	0.037	300^f)^	−	−	−	[160]
**Ru‐35** ^b)^	7	0.05	370^f)^	−	−	−	[160]

^a)^ 0.216 mM catalyst with 0.4 M CAN in aqueous triflic acid (TfOH);

^b)^ 1 mM catalyst in aqueous triflic acid (pH 1) containing 20 vol% MeCN (ionic strength = 0.5 M);

^c)^ 1 mM catalyst in pH 7 phosphate buffer containing 20 vol% MeCN (ionic strength = 0.5 M);

^d)^ Phosphate buffer solutions;

^e)^ TON at 1 h;

^f)^ TON at 24 h;

^g)^ Values of the overpotential (*E*_cat/2_) extracted from the CVs recorded at pH 7. WNA: water nucleophilic attack. I2M: bimolecular radical–radical coupling. RHE: reversible hydrogen electrode.

### 4.4 Nitrogen Reduction Reaction (NRR)

**Figure 35:**
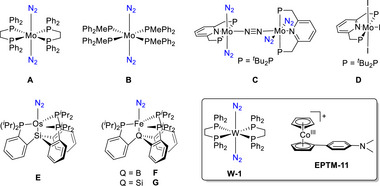
Molecular nitrogen reduction reaction (NRR) catalysts were explored in combination with **EPTM‐11** under electrocatalytic conditions.^[^ ^169^ ^]^

**Figure 36:**
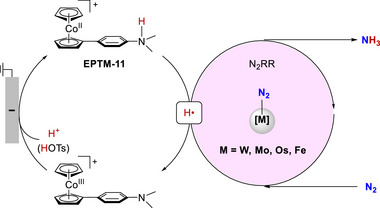
Tandem catalysis based on coupling a PCET mediator (**EPTM‐11**) with molecular NRR catalysts to enable well‐defined electrocatalysis at comparatively mild potentials (−1.2 V using *p*‐toluenesulfonic acid (TsOH)).^[^ ^169^ ^]^

Peters et al. introduced a tandem electrocatalytic approach for the NRR that couples N_2_‐activating catalysts with a PCET mediator (**Figure** **35**, **W‐1** and **EPTM‐11**).^[^ ^169^ ^]^ From a thermodynamic standpoint, the incorporation of **EPTM‐11** is essential to initiate catalysis under mild electrochemical conditions (**Figure** **36**, −1.2 V vs Fc^+/0^). In the absence of **EPTM‐11**, **W‐1** did not promote the formation of NH_3_ under the same electrochemical potential. In addition, a standalone Fe‐based NRR catalyst had to operate at a potential of −2.1 V vs Fc^+/0^, as previously reported by the same group. In contrast, **EPTM‐11** enabled productive electrocatalysis (11.3 ± 0.5 equiv. NH_3_ per **W‐1**, FE = 44.5%) (**Table** **17**). The corresponding free energy input for the NRR (ΔΔ*G*_f_ ≈ 36.5 kcal mol^−1^) represents an energetic demand at least 50 kcal mol^−1^ lower than those of most chemically driven protocols, which underscores the thermodynamic efficiency of the PCET‐mediated pathway. In summary, this cooperative tandem strategy provides notable thermodynamic advantages, thereby bypassing the energetically costly stepwise ET or PT and suppressing the competing hydrogen evolution reaction (HER). This work demonstrates how EPTMs can expand the scope of the NRR, providing a biomimetic pathway that more closely resembles enzymatic nitrogen fixation.

**Table 17:** Performances of different molecular catalysts in the electrochemical NRR.^[^ ^169^ ^]^

Catalyst	Equiv. NH_3_/Catalyst	FE [%]
**W‐1**	11.3 ± 0.5	44.5 ± 1.9
**W‐1** ^a)^	40	43
**A**	13	51
**B**	14	55
**C**	8.7	34
**D**	<0.1	<1.0
**E**	5.6	22
**F**	4.5	18
**G**	1.5	4.5

General condition: CPE at −1.35 V vs Fc^+/0^ in 0.1 M [Li][NTf_2_] solution in 1,2‐dimethoxyethane (DME) containing 0.05 mM **EPTM‐11**, 0.05 mM NRR catalyst, and 5 mM TsOH using a boron‐doped diamond plate working electrode.

a^)^ GC foam was used as the working electrode instead, and the concentration was 0.01 mM for both co‐catalysts and 1 mM for TsOH.

### 4.5 Oxidative Organic Transformations (OOTs)

#### 4.5.1 EPTMs in Molecular Catalyst–Promoted Electrochemical OOTs

**Figure 37:**
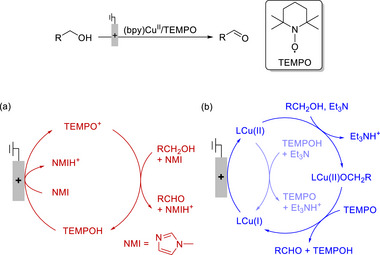
Proposed mechanisms for the electrochemical oxidation of alcohols catalyzed by a) 2,2,6,6‐tetramethyl‐1‐piperidinyloxyl (TEMPO)‐only and b) (bpy)Cu^II^/TEMPO systems. Reproduced with permission.^[^ ^44^ ^]^ Copyright 2020, American Chemical Society.

Stahl et al. (2016) reported the electrochemical oxidation of alcohols mediated by (bpy)Cu^II^/2,2,6,6‐tetramethyl‐1‐piperidinyloxyl (TEMPO) (**Figure** **37**).^[^ ^43^ ^]^ Under most conditions, the substrate scope of the TEMPO‐promoted electrochemical alcohol oxidation reaction was limited. The poor tolerance of substrate functional groups was due to the excessively high oxidation potential required to regenerate TEMPO^+^ from TEMPOH (Figure 37a). In the (bpy)Cu^II^/TEMPO system, the role of (bpy)Cu^II^ was disclosed by UV–vis studies: in the presence of 2,6‐lutidine as a Lewis base, TEMPO could be readily formed from TEMPOH, and benzyl alcohol underwent anaerobic oxidation. This outcome implied that the (bpy)Cu^II^‐alkoxide intermediate was generated and exhibited thermodynamic feasibility in the reaction with TEMPO via a PCET step to afford the organic product (Figure 37b). Notably, the *E*_CAT_ is at the (bpy)Cu^II/I^ potential, exhibiting a cathodically shift by 0.5 V relative to the TEMPO‐only system (**Table** **18**), which could improve compatibility with diverse functional groups and expand the substrate scope. Compared with the TEMPO‐only system, (bpy)Cu^II^/TEMPO demonstrated a five‐fold higher TOF at a notably lower *E*_CAT_ (Table 18). Although the TEMPO‐only and (bpy)Cu^II^/TEMPO systems followed a second‐order rate law, the former mediated alcohol oxidation via a 2e^−^ pathway (Figure 37a), whereas a 1e^−^ + 1e^−^ route was operational in the latter case (Figure 37b).

**Table 18:** Performances of (bpy)Cu^II^/TEMPO and TEMPO‐only systems in the electrochemical oxidation of alcohols.^[^ ^44^ ^]^

Catalyst system	(bpy)^II^Cu/TEMPO^a)^	TEMPO^b)^
Kinetic order with respect to (bpy)^II^Cu	1st^c)^	1st^e)^
Kinetic order with respect to benzyl alcohol	1st^d)^	1st^f)^
Potential of current plateau (vs Fc^+/0^)	−0.14 V	0.36 V
*k*_obs_ (20 mM PhCH_2_OH)	11.6 s^−1^	2.3 s^−1^

^a)^ 0.1 M NBu_4_ClO_4_ in MeCN, *v*: 150 mV s^−1^;

^b)^ 0.1 M NBu_4_ClO_4_ in MeCN, *v*: 150 mV s^−1^;

^c)^ 5 mM TEMPO, 50 mM Et_3_N, 100 mM PhCH_2_OH ;

^d)^ 1 mM (bpy)Cu(I), 5 mM TEMPO, 50 mM Et_3_N ;

^e)^ 450 mM *N*‐methylimidazole (NMI), 100 mM PhCH_2_OH;

^f)^ 1 mM TEMPO, 450 mM NMI.

**Figure 38:**
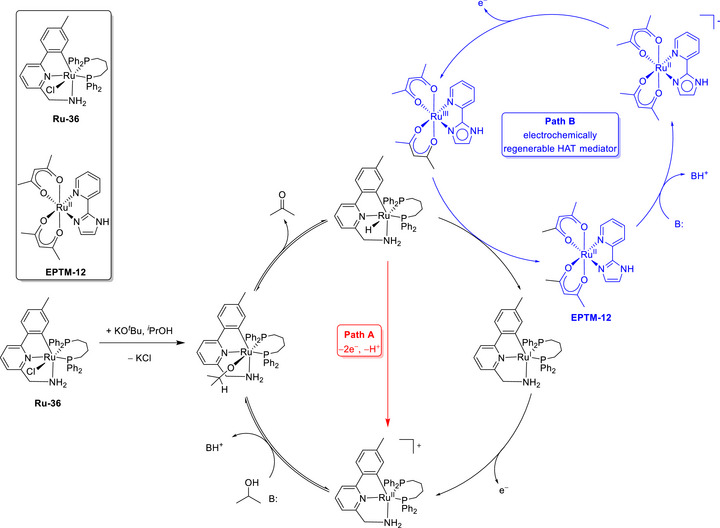
Proposed mechanisms for the electrochemical oxidation of alcohols catalyzed by the **Ru‐36/EPTM‐12** system. Reproduced with permission.^[^ ^170^ ^]^ Copyright 2020, American Chemical Society.

Waymouth et al. demonstrated that HAT is critically involved in the electrocatalytic oxidation of alcohols.^[^ ^170^ ^]^ Conventional ET/PT pathways for regenerating metal hydrides are kinetically demanding, whereas an electrochemically regenerable HAT mediator circumvents this barrier. In the study of electrochemical isopropanol oxidation, **Ru‐36** alone showed TOFs of 0.6 and 3.2 s^−1^ with *^t^* Bu‐P_4_ and KO*^t^* Bu as Lewis bases, respectively, at *E*_CAT_ = −0.60 V vs Fc^+/0^ (**Table** **19**). The addition of **EPTM‐12** to **Ru‐36** resulted in the establishment of a new 1e^−^ + 1e^−^ pathway, whereas the sole‐catalyst system followed an inefficacious 2e^−^ process (**Figure** **38**, Path B vs A). This cooperation lowered*η* by ∼450 mV (*E*_CAT_: −1.05 V vs Fc^+/0^) and thereby enhanced catalytic efficiency, albeit at the cost of lower TOFs (Table 19). CPE at –1.05 V vs Fc^+/0^ for 4 h with **Ru‐36** (0.50 mM), isopropanol (0.5 M), KO*^t^* Bu (30 mM), and **EPTM‐12** (1.5 mM) generated 0.023 mmol acetone, as determined by ^1^H NMR spectroscopy. This result corresponds to a TON of 4 with an FE of ∼85%, whereas only background current was observed in the absence of **EPTM‐12** under identical conditions. These results highlight the utility of HAT mediators for enabling efficient OOTs.

**Table 19:** Performances of **Ru‐36/EPTM‐12** in an electrochemical oxidative organic transformation (OOT).^[^ ^170^ ^]^

Catalyst/EPTM	Onset (V vs Fc^+/0^)	*E*_cat/2_ (V vs Fc^+/0^)	TOF [s^−1^]^g)^
**Ru‐36**	−1.0^a)^	−0.85^a)^	0.6^c)^ / 3.2^d)^
**Ru‐36/EPTM‐12**	−1.4^b)^	−1.3^b)^	0.3^e)^ / 0.7^f)^

^[a]^ Determined from the CV curves of 1 mM **Ru‐36** in 0.1 M NBu_4_BF_4_ in tetrahydrofuran (THF) with 0.30 M isopropanol, 1.1 mM KO*^t^* Bu, and 70 mM *^t^* Bu‐P_4_ (CAS #111324‐04‐0);

^b)^ Determined from the CV curves of 1 mM **Ru‐36** in 0.1 M NBu_4_BF_4_ in THF with 0.30 M isopropanol, 1.1 mM KO*^t^* Bu, 70 mM *^t^* Bu‐P_4_, and 1.3 mM **EPTM‐12**;

^c)^ 1 mM **Ru‐36** in the presence of 70 mM *^t^* Bu‐P_4_, and 0.50 M isopropanol;

^d)^ 1 mM **Ru‐36** in the presence of 300 mM KO*^t^* Bu, and 0.50 M isopropanol;

^e)^ 1 mM **Ru‐36** in the presence of 1.3 mM **EPTM‐12**, 70 mM *^t^* Bu‐P_4_, and 0.50 M isopropanol;

^f)^ 1 mM **Ru‐36** in the presence of 1.3 mM **EPTM‐12**, 300 mM KO*^t^* Bu, and 0.50 M isopropanol;

^g)^ Calculated using Equations (7) and (8). *v* = 25 mV s^−1^.

**Figure 39:**
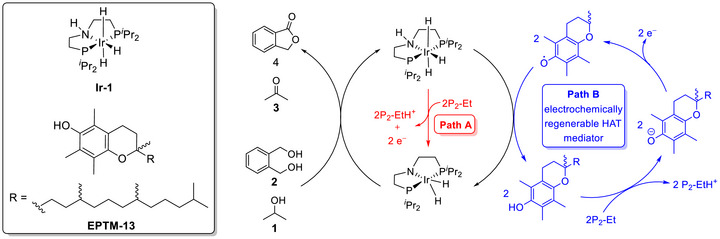
Proposed mechanisms for the electrochemical oxidation of alcohols catalyzed by **Ir‐1**/**EPTM‐13**. Reproduced with permission.^[^ ^171^ ^]^ Copyright 2020, American Chemical Society.

The same team also showed that electron‐rich phenoxyl mediators could function as the EPTM (**EPTM‐13**), enabling electrocatalytic HAT between the molecular catalyst (**Ir‐1**) and substrate with a low‐energy barrier route.^[^ ^171^ ^]^ In this study, the direct electrooxidation of **Ir‐1** required *E*_onset_ = −0.65 V vs Fc⁺^/0^, whereas **EPTM‐13** (**Figure** **39**) lowered *E*_onset_ to −1.07 V vs Fc⁺^/0^, reducing *E*_CAT_ by 420 mV. CV measurements subsequently revealed a TOF of up to 14.6 s^−1^ in THF. CPE experiments demonstrated that **Ir‐1/EPTM‐13** promoted the oxidative transformation of isopropanol (**1**) to acetone (**3**) at −735 mV vs Fc⁺^/0^ with 93% FE; however, no activity was observed without **EPTM‐13** at this potential (**Table** **20**). **Ir‐1** promoted the electrocatalytic oxidation of **1** to **3** at −355 mV with a modest FE of 78%, i.e., at a potential 450 mV anodically than that of the **Ir‐1/EPTM‐13** system (Table 20). Mechanistic studies showed that **EPTM‐13** mediated the regeneration of active Ir species through two sequential HATs, in contrast to the unfavorable HAT pathway observed for 2 equiv. P_2_‐Et (Figure 39, Path B vs A). Kinetic isotope investigations and comparisons with established systems supported the conclusion that the initial homolysis of an Ir–H bond is the TLS.

**Table 20:** Performance of **Ir‐1/EPTM‐13** in an electrochemical OOT.^[^ ^171^ ^]^

Entry	Mediator (mM)	Substrate	Product	Potential (mV vs Fc^+/0^)	FE%
1	0	**1**	**3**	−355	78
2	2.5	**1**	**3**	−735	93%
3	0	**1**	**3**	−735	–
4	2.5	**2**	**4**	−735	100%

General condition: 3.1 mM **Ir‐1**, 63 mM P_2_‐Et (CAS #165535‐45‐5), 100 mM NBu_4_BF_4_ as the supporting electrolyte in the THF solution and 25 °C. Divided glass H‐cell with a reticulated vitreous carbon working electrode.

#### 4.5.2 EPTMs in Molecular Catalyst–Promoted Chemical OOTs

**Figure 40:**
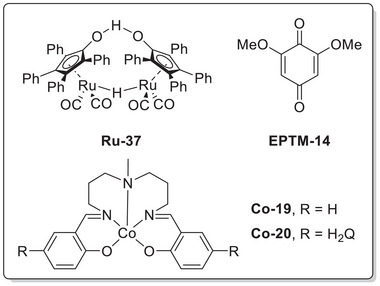
Structures of the catalysts and EPTM used in Bäckvall's work.^[^ ^42^, ^172^, ^173^, ^174^ ^]^

Inspired by the biological respiratory chain, the NAD^+^/NADH redox couple can be used to bypass the kinetically sluggish pathway of the aerobic oxidation of organic substrates by O_2_ or H_2_O_2_. Bäckvall et al. demonstrated that mediators such as **EPTM‐9**/**EPTM‐10** derivatives or Co(sal‐type)‐H_2_Q hybrids (**Figure** **40**) can promote ET/PT in the metal‐catalyzed aerobic oxidation of organic compounds, thereby increasing the related yields and conversions.^[^ ^42^, ^172^ ^]^ The dehydrogenative oxidation of 1‐phenylethanol (**5**) in the presence of a Ru catalyst (**Ru‐37**), 2,6‐dimethoxy‐1,4‐benzoquinone (**EPTM‐14**), and Co(salmdpt, salmdt: bis[3‐(salicylideneimino)propyl]methylamine) (**Co‐19**) proceeded with a 100% conversion after 24 h at a low catalyst loading of 0.5 mol% (**Table** **21**, entry 1).^[^ ^173^ ^]^ The efficiency of this reaction was further optimized under milder conditions using **Co‐19** with a hybrid EPTM featuring two H_2_Q units (**Co‐20**). The quantitative conversion of **5** to **6** with **Ru‐37**/**Co‐20** was achieved in 9−10 h (Table 21, entries 2 and 3).^[^ ^174^ ^]^

**Table 21:** Performance of **Ru‐37** with different mediators in aerobic alcohol oxidation.^[^ ^173^, ^174^ ^]^

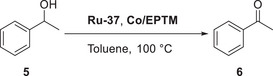				
Entry	Catalyst [mol%]	Solvent, temperature	Time [h]	Conversion [%]^c)^
1^a)^	**Ru‐37** (0.5 mol%) **EPTM‐10** (20 mol%) **Co‐19** (2 mol%)	Toluene, 100 °C	24	100
2^b)^	**Ru‐37** (0.5 mol%) **Co‐19** (1 mol%)	Neat, 75 °C	9	100
3^b)^	**Ru‐37** (0.5 mol%) **Co‐20** (1 mol%)	MeCN, 75 °C	10	100

^a)^ The reaction was carried out on a 1 mmol scale in 5 mL of solvent using a balloon filled with ∼ 2% O_2_ in N_2_;

^b)^ The reaction was carried out on a 2 mmol scale in 1 mL of solvent under air;

^c)^ Conversion was determined using gas chromatography.

**Figure 41:**
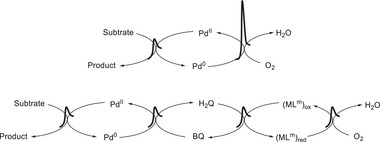
Influence of EPTMs on the energy barrier of a Pd^II^‐catalyzed aerobic oxidation reaction. L^m^: macrocyclic ligand. Reproduced with permission.^[^ ^42^ ^]^ Copyright 2008, Wiley‐VCH.

The concept of hybrid EPTMs is also applicable to the oxidative 1,4‐diacetoxylation of **7** with Pd(OAc)_2_ as a co‐catalyst, as examined by Bäckvall et al.^[^ ^175^ ^]^ As shown in **Table** **22**, a hybrid Co(sal)–H_2_Q catalyst (**Co‐2**) achieved an 80% isolated yield of **8** in 7 h, whereas a mixture of Co(sal) (**Co‐1**) and H_2_Q (**EPTM‐9**) afforded a similar yield of **8** in 28 h. Additionally, Bäckvall et al. (2020) reported a stereoselective carbocyclization of **9** to **10** co‐catalyzed by Pd(OAc)_2_ and **Co‐2** (**Table** **23**).^[^ ^176^ ^]^ Compared with the **Co‐1**/**EPTM‐9** and **Co‐1/EPTM‐10** systems, **Co‐2** afforded a higher yield and conversion under the same conditions. The same group demonstrated that **Co‐2** could mediate different types of carbocyclization (**Table** **24**, 11 to 12), showing higher efficiency than the **Co‐1**/**EPTM‐9** and **Co‐1**/**EPTM‐10** systems.^[^ ^177^ ^]^ Overall, these results suggested that the reoxidation of Pd^0^ to Pd^II^ by the hybrid EPTMs had a lower kinetic barrier than the direct oxidation of Pd^0^ with molecular O_2_ as the terminal oxidant (**Figure** **41**). Importantly, the utilized EPTMs facilitated PT to the terminal oxidant (O_2_) and afforded H_2_O instead of H_2_O_2_ as the exclusive product, preventing the H_2_O_2_‐induced deactivation of the Pd catalyst or product decomposition.

**Table 22:** Performances of different catalysts for the coupled aerobic 1,4‐diacetoxylation of 1,3‐cyclohexadiene (**7**).^[^ ^175^ ^]^

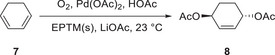				
Entry^a^ ^)^	Catalyst/ EPTM	10 × initial rate of O_2_ consumption [mL h^−1^]	Time [h]	Yield [%]^b)^
1	**Co‐1 + EPTM‐9**	1.0 ± 0.2	28	70
2	**Co‐2**	4.2 ± 0.8	7	80

^a^ ^)^ **7** (0.22 mmol), **Co‐1** (0.011 mmol) + **EPTM‐9** (0.022 mmol) or **Co‐2** (0.011 mmol), Pd(OAc)_2_ (0.011 mmol), lithium acetate dihydrate (0.56 mmol), and acetic acid (1 mL) at 25 °C. The results represent the averages of at least two trials;

^b)^ Yields were determined by ^1^H nuclear magnetic resonance (NMR) spectroscopy.

**Table 23:** Effects of different EPTMs on the reaction of **9** with PhBneo to afford **10**.^[^ ^176^ ^]^

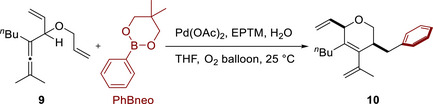				
Entry^a)^	Catalyst	EPTM	Time [h]	Yield [%]
1	**Co‐1**	**EPTM‐9**	9	1
2	**Co‐1**	**EPTM‐10**	9	9
3	**Co‐2**	−	9	25
4	**Co‐1**	**EPTM‐9**	16	5
5	**Co‐1**	**EPTM‐10**	16	13
6	**Co‐2**	−	16	39
7	**Co‐1**	**EPTM‐9**	24	9
8	**Co‐1**	**EPTM‐10**	24	18
9	**Co‐2**	−	24	48
10	**Co‐1**	**EPTM‐9**	40	15
11	**Co‐1**	**EPTM‐10**	40	24
12	**Co‐2**	−	40	57

^a)^ Unless otherwise noted, the reactions were conducted at 25 °C in THF (0.5 mL) with **9** (0.1 mmol), PhBneo (0.13 mmol), and H_2_O (0.1 mmol) in the presence of Pd(OAc)_2_ (5 mol%) and EPTM (10 mol%) under O_2_ (O_2_‐filled balloon).

**Table 24:** Performances of different EPTMs for the borylative carbocyclization of bisallene **11**.^[^ ^177^ ^]^

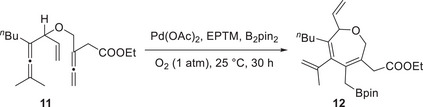		
Entry^a)^	Catalyst/EPTM	Yield [%]^b)^
1	None	0^c)^
2	20 mol% **EPTM‐10**	15
3	10 mol% **Co‐1**, 20 mol% **EPTM‐10**	48
4	10 mol% **Co‐1**, 20 mol% **EPTM‐9**	35
5	10 mol% **Co‐2**	78^d)^
6	100 mol% **EPTM‐10**	78^d)^

^a^ ^)^Unless otherwise noted, the reaction was conducted using **11** (0.1 mmol, 1.0 equiv.), B_2_pin_2_ (0.13 mmol, 1.3 equiv.), Pd(OAc)_2_ (5 mol%), and EPTMs (10–20 mol%) in 0.1 M acetone under O_2_ (1 atm) at 25 °C for 30 h;

^b)^ Yields were determined by ^1^H NMR spectroscopy using anisole as an internal standard;

^c)^ 93% of **7** was recovered;

^d)^ <1% of **7** was recovered.

**Figure 42:**
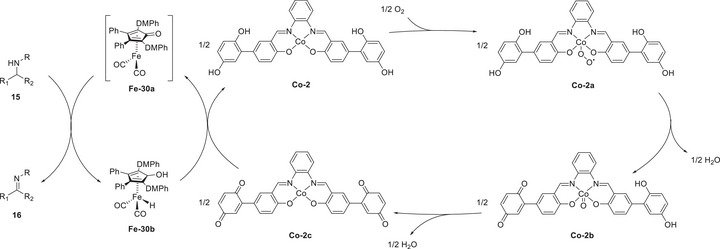
Biomimetic oxidation approach using an Fe complex (**Fe‐30**) as a substrate‐selective redox catalyst and a hybrid hydroquinone/Co catalyst (**Co‐2**) as the EPTM. Reproduced with permission.^[^ ^179^ ^]^ Copyright 2021, Wiley‐VCH.

Recently, Bäckvall et al. (2020) have examined the Fe^II^‐catalyzed biomimetic aerobic oxidation reaction for the first time, showing that the corresponding catalytic cycle is analogous to that of the respiratory chain.^[^ ^178^ ^]^ The oxidation of an alcohol (**13**) to a ketone (**14**) by molecular O_2_ was facilitated by the combination of **Co‐19**/**EPTM‐14** with **Fe‐29** or **Fe‐30** as the catalyst (**Table** **25**). Compared with **Fe‐29**, the co‐catalytic system containing **Fe‐30** effectively promoted the oxidation of primary and secondary alcohols to the corresponding aldehydes or ketones under aerobic conditions. The same group realized the catalytic aerobic oxidation of an amine (**15**) to an aldimine (**16**) using a structurally similar Fe catalyst under aerobic conditions (**Figure** **42**).^[^ ^179^ ^]^ The utilization of a hybrid Co catalyst as an EPTM (**Co‐2**) increased the yield of **16** from trace amounts to 73% (**Table** **26**). The slower transfer of electrons between O_2_ and **Co‐19/EPTM‐14** compared with that between O_2_ and **Co‐2** accounted for the poor yield observed in the former case, where **Fe‐30** was gradually deactivated during the reaction. Notably, **Fe‐29** and **Fe‐30**, rather than EPTMs, served as HAT catalysts and directly dehydrogenated the alcohol substrates (Tables 25 and 26). Nonetheless, in the aforementioned Pd^II^‐catalyzed aerobic oxidation reaction (Tables 21, 22, 23, 24), EPTMs facilitated PT to the terminal O_2_ instead of the substrate. These studies highlight the versatile roles of EPTMs in OOTs.

**Table 25:** Performance of the **Co‐19**/**EPTM‐14** system in the Fe‐catalyzed biomimetic oxidation of alcohol **13**.^[^ ^178^ ^]^

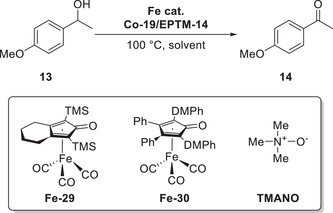				
Entry^a)^	Catalyst	Co‐19 (mol%)	EPTM‐14 (equiv.)	Yield (%)^b)^
1^c)^, ^d)^	**Fe‐29**	−	1.2	26
2^c)^, ^e)^	**Fe‐29**	−	1.2	8
3	**Fe‐29**	40	1.2	47
4	‐	40	1.2	3
5^f)^	**Fe‐30**	4	0.4	71
6^g)^	**Fe‐30**	4	0.4	>95
7^g)^, ^h)^	**Fe‐30**	4	0.4	>95

^a)^ The reaction was conducted under air at 100 °C using 0.5 mmol **13**, Fe catalyst (10 mol%), TMANO (10 mol%), and anisole (3 mL);

^b)^ Yields after 1 h were determined using gas chromatography;

^c)^ Solvent replacement with toluene;

^d)^ Ar atmosphere;

^e)^ O_2_ atmosphere;

^f)^ Addition of 4 Å molecular sieves;

^g)^ Higher concentration (0.33 M **13**, 1.5 mL anisole);

^h)^ Addition of 1 equiv. K_2_CO_3_.

**Table 26:** Performances of **Co‐19**/**EPTM‐14** and **Co‐2** systems in the Fe‐catalyzed biomimetic oxidation of amine **15**.^[^ ^179^ ^]^

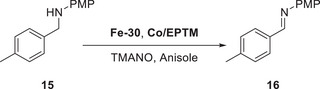			
Entry	Solvent	Co/EPTM	Yield [%]^e)^
1^a)^	Anisole	**Co‐19/EPTM‐14**	Trace
2^b)^	Anisole	**Co‐2**	24
3^b)^, ^c)^	Methanol	**Co‐2**	41
4^d)^	Methanol	**Co‐2**	69
5^d)^	Methanol/1,4‐dioxane (1:1)	**Co‐2**	73

^[a]^ **Co‐19** (4 mol%), **EPTM‐14** (40 mol%), **Fe‐30** (20 mol%), and TMANO (20 mol%) at 100 °C;

^b)^ **Co‐2** (20 mol%), **Fe‐30** (10 mol%), and TMANO (10 mol%) at 80 °C;

^c)^ The reaction was performed at 40 °C;

^d)^ **Co‐2** (10 mol%), **Fe‐30** (5 mol%), and TMANO (5 mol%) at 60 °C;

^e)^ The yield after 2 h was determined by ^1^H NMR spectroscopy using 1,3,5‐trimethoxybenzene as an internal standard. PMP = *p*‐methoxyphenyl.

**Figure 43:**
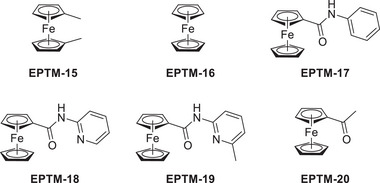
Electrocatalytic PCET mediators developed by Mondal et al., **EPTM‐*n*** (***n*** = **15**–**20**).^[^ ^180^ ^]^

**Figure 44:**
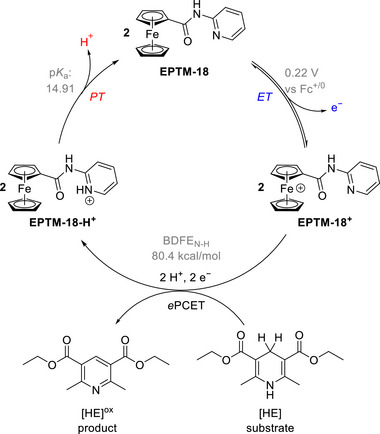
Calculated and measured thermodynamic parameters for the **EPTM‐18**‐catalyzed electrochemical Hantzsch ester (HE) oxidation. Reproduced with permission.^[^ ^180^ ^]^ Copyright 2024, American Chemical Society.

**Figure 45:**
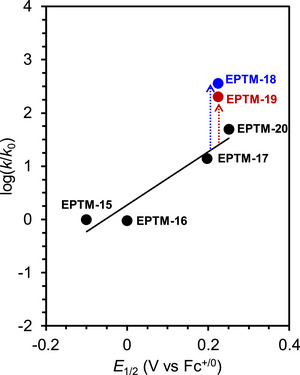
Performances of **EPTM‐*n*** (***n*** = **15**–**20**) systems in electrochemical HE oxidation. Reproduced with permission.^[^ ^180^ ^]^ Copyright 2024, American Chemical Society.

Mondal et al. (2024) utilized ferrocene derivatives as mediators (**Figure** **43**, EPTM‐15–20) for catalyzing the oxidation of Hantzsch ester (HE).^[^ ^180^ ^]^ **EPTM‐18** comprises a covalently attached NADH‐analogous subunit capable of being an electrocatalytic PCET (*e*PCET) mediator. The intramolecular PT and ET sites of **EPTM‐18** increased the adaptability of the mediator in terms of the redox potential and BDFE of N−H bond (BDFE_N−H_). Notably, the BDFE_N−H_ of **EPTM‐18** (∼80 kcal mol^−1^) was sufficient to promote the 2H^+^/2e^−^ electrochemical oxidation of HE (**Figure** **44**, BDFE_C−H_ ≈ 70 kcal mol^−1^). The effectiveness of **EPTM‐18** and **EPTM‐19** was demonstrated by the breakage of the log(*k*_cat_)–*E*_1/2_ relationships of the Fc‐based mediators (**Table** **27** and **Figure** **45**). This result underscores the significance of *e*PCET in facilitating electrochemical organic reactions, thereby paving the way for innovative and sustainable solutions.

**Table 27:** Performances of **EPTM‐*n*** (***n*** = **15**–**20**) systems in electrochemical HE oxidation.^[^ ^180^ ^]^ ^a^ ^)^

Mediator	*E*_1/2_ [mV]	Log (*k*/*k*_0_)^b)^
**EPTM‐15**	−0.1	0
**EPTM‐16**	0.0	−0.02
**EPTM‐17**	0.197	1.15
**EPTM‐18**	0.251	1.7
**EPTM‐19**	0.225	2.3
**EPTM‐20**	0.225	2.55

^a)^ Supporting electrolyte = 100 mM [NBu_4_F] in MeCN; glassy carbon, Pt wire, and Ag/AgCl were used as the working, counter, and reference electrodes, respectively; 298 K; *v* = 5 mV s^−1^; potential adjusted with respect to Fc^+/0^;

^b)^ *k* and *k*_0_ are the reaction rate constants in the presence and absence of a mediator, respectively.

### 4.6 Reductive Organic Transformations (ROTs)

#### 4.6.1 EPTMs in Molecular Catalyst–Promoted Electrochemical ROTs

Reductive CPET for the electrochemical reduction of C–C π‐bonds remains underdeveloped, particularly in the case of activated alkenes, which are susceptible to side reactions such as the HER or oligomerization. Peters et al. demonstrated that the C–C π‐bonds of fumarate esters (**17** and **18**) can be selectively hydrogenated via *e*CPET using a cobaltocene‐based mediator linked to a Brønsted base substituent, namely *N*,*N*‐dimethylaniline (**Table** **28**, **EPTM‐11**).^[^ ^181^ ^]^ This strategy enabled reductive *e*CPET, resulting in net hydrogenation (2*e* ^−^/2H^+^) at −1.30 V vs Fc^+/0^. In the absence of **EPTM‐11**, the reaction did not proceed efficiently, and **19** and **20** were obtained in low yields.

**Table 28:** Performances of **EPTM‐11** in the electrochemical reduction of diphenyl and dicyclohexyl fumarates.^[^ ^181^ ^]^ ^a)^

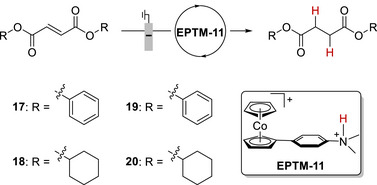				
Substrate	EPTM‐11 [mM]	Product	Time [h]	Yield [%]
**17**	−	**19**	20	24
**17**	1	**19**	20	86
**18**	−	**20**	48	2
**18**	2	**20**	48	78

General conditions: 50 mM substrate, 100 mM TfOH, and 200 mM [NBu_4_PF_6_] in DME.

^a)^ CPE with TsOH in an undivided cell at −1.30 V vs Fc^+/0^.

#### 4.6.2 EPTMs in Molecular Catalyst–Promoted Photochemical ROTs

**Figure 46:**
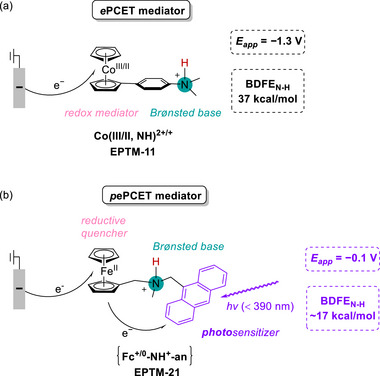
Comparison of a) an electrocatalytic PCET mediator (**EPTM‐11**) with b) a photoelectrocatalytic PCET mediator (**EPTM‐21**). Reproduced under terms of the CC‐BY 4.0 license.^[^ ^185^ ^]^ Copyright 2024, American Chemical Society.

**Figure 47:**
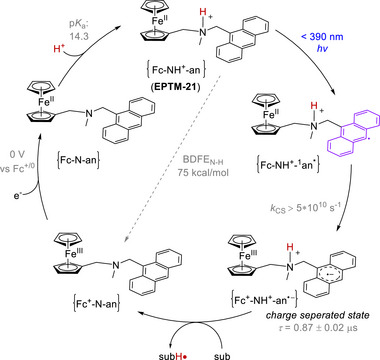
Calculated and measured thermodynamic parameters of **EPTM‐21**‐mediated ground‐ and excited‐state PCET. *k*_CS_ denotes the charge‐separated rate constant. Reproduced under terms of the CC‐BY 4.0 license.^[^ ^185^ ^]^ Copyright 2024, American Chemical Society.

In photocatalysis, excited‐state PCET allows ET or PT steps to occur via a concerted mechanism, avoiding the formation of high‐energy charged intermediates. The excited state of a PS typically has a redox potential markedly different from that of the ground state, which enables reactions thermodynamically challenging or inaccessible under ambient conditions.^[^ ^182^, ^183^, ^184^ ^]^ Peters et al. (2024) utilized a PS with an Fc linkage as a photoelectrocatalytic PCET (*pe*PCET) mediator to catalyze an ROT (**Figure** **46**a, **EPTM‐21**).^[^ ^185^ ^]^ Compared with the cobaltocene‐based *e*PCET mediator reported by the same group (Figure 46b, **EPTM‐11**),^[^ ^181^ ^]^ **EPTM‐21** mediated the reductive reaction at a milder applied potential (−0.1 vs −1.3 V vs Fc^+/0^) and featured a substantially weaker excited‐state BDFE_N−H_ (Figure 46, 17 vs 37 kcal mol^−1^). This excited‐state BDFE_N−H_ (17 kcal mol^−1^ for **EPTM‐18**) corresponds to the ground‐state BDFE_N−H_ of 75 kcal mol^−1^ (**Figure** **47**). The substrate scope of the **EPTM‐21** system was further examined using photochemical and photoelectrochemical approaches (*p*PCET and *pe*PCET, respectively), as shown in **Table** **29**. The reactivity of **EPTM‐21** under photochemical conditions, along with the results of a light on/off experiment, indicated that the excited‐state form of **EPTM‐21**, {Fc^+^‐NH^+^‐an^•−^}, was involved in mediating the reductive reactions of **21**−**23** via PCET pathways (Figure 47).

**Table 29:** Performances of **EPTM‐21** in the photochemical reduction of organic substrates.^[^ ^185^ ^]^

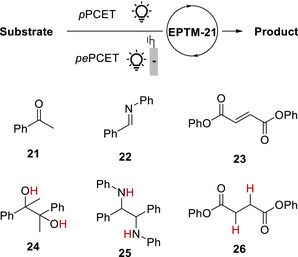					
Substrate	BDFE_X‐H_ [kcal mol^−1^]	Product	Yield (*p*PCET)	Yield (*pe*PCET)	TON^a)^
**21**	36	**24**	29%	36 ± 4%	18 ± 2
**22**	50	**25**	65%	30%	15
**23**	45	**26**	42%	9%	9

^a)^ TON was determined after 24 h at *E*_app_ = −0.1 V vs Fc^+/0^ under 390 nm irradiation, and TOF was calculated as TON/86 400 s.

## 5 Summary and Outlook

The integration of EPTMs, RMs, and PRs as co‐catalysts into homogeneous catalytic systems is a notable advancement in the development of efficient molecular photo‐ and electrocatalysts for energy‐related small‐molecule transformations and sustainable organic reactions. This review systematically summarizes recent achievements in this field. It provides valuable insights into the catalytic performance enhancements enabled by these three strategies, focusing on their roles in modulating the reaction kinetics (e.g., *k*_cat_ and TOF_max_), thermodynamics (e.g., *η* and BDE), and selectivity. Unsurprisingly, in these dual‐catalytic systems, the conventional trade‐off between log(kinetics) and thermodynamics can be circumvented, as revealed by LFER analysis. The presented LFER data provide a quantitative understanding of the interplay between molecular catalysts and co‐catalysts from a molecular perspective.

The reviewed studies highlight several key advancements. Biomimetic approaches inspired by the hydrogenase structure provide valuable guidelines for designing PRs. Based on the active sites of hydrogenases, synthetic catalysts featuring strategically positioned PRs may exhibit superior activities and selectivities. To date, PRs have been comprehensively implemented in small‐molecule catalysts, such as those promoting the CO_2_RR, ORR, and WOR. Enhancing catalyst performance with the aid of PRs is feasible when protonation is the TLS. Similarly, the integration of RMs into homogeneous catalysts illustrates the means by which synthetic RMs emulate the ET processes observed in natural systems. RMs facilitate electron transport between catalysts and electrodes, or within the catalytic systems themselves, thereby helping to avoid the formation of high‐energy intermediates and enhancing catalytic turnover. For redox reactions involving multiple proton–electron transfers, EPTMs may change the reaction mechanism from stepwise PCET to CPET with a low energy barrier. The reaction selectivity can also be modulated using EPTMs. Overall, PR‐, RM‐, and EPTM‐based systems offer the benefits of tunable catalytic pathways, reduced energy barriers, and improved product selectivities across diverse reactions, including CO_2_ and O_2_ reduction, N_2_ fixation, and H_2_O oxidation, as well as other organic transformations.

Despite these advancements, several challenges remain. For example, a precise mechanistic understanding and quantification of the mediator effects under various conditions are required. In this context, the spatial configuration of the PR relative to the catalytic site must be precisely controlled to effectively shuttle protons. The interplay between the mediator redox potential and catalytic potential is critical for these co‐catalytic systems and must be comprehensively examined in different media. For EPTM‐containing systems, the mutual influence of the ET tendency and p*K*_a_ values of different protonation sites must be considered for different redox catalysts. Additionally, ensuring the stability and longevity of these integrated catalytic systems under operating conditions remains a significant synthetic and practical challenge.

Future research should more comprehensively investigate the interplay between the kinetic and thermodynamic parameters modulated by EPTMs, RMs, and PRs using LFER analysis. Computational modeling combined with advanced spectroscopic and electrochemical analyses could help unravel the cooperative interactions within these integrated systems. Additionally, expanding the library of stable and robust EPTMs, RMs, and PRs through systematic ligand and scaffold design could enhance the applicability of these catalysts across a broader range of conditions. Ultimately, continued innovations in the strategic design and thermochemistry of EPTM, RM, and PR systems could yield highly efficient and sustainable catalysts. These advances are indispensable for addressing global energy challenges and facilitating the transition toward a sustainable and environmentally responsible chemical industry.

## Conflict of Interest

The authors declare no conflict of interest.

## References

[1] A. I. Osman , L. Chen , M. Yang , G. Msigwa , M. Farghali , S. Fawzy , D. W. Rooney , P.‐S. Yap , Environ. Chem. Lett 2023, 21, 741. 10.1007/s10311-022-01435-8 PMC8992416 35431715

[2] T. M. Lenton , C. Xu , J. F. Abrams , A. Ghadiali , S. Loriani , B. Sakschewski , C. Zimm , K. L. Ebi , R. R. Dunn , J.‐C. Svenning , M. Scheffer , Nat. Sustain. 2023, 6, 1237.

[3] P. Andre , T. Boneva , F. Chopra , A. Falk , Nat. Clim. Chang. 2024, 14, 253.

[4] T.‐Z. Ang , M. Salem , M. Kamarol , H. S. Das , M. A. Nazari , N. Prabaharan , Energy Strategy Rev. 2022, 43, 100939.

[5] J. Jurasz , F. Canales , A. Kies , M. Guezgouz , A. Beluco , Sol. Energy 2020, 195, 703.

[6] R. Schlögl , Top. Catal. 2016, 59, 772.

[7] A. Bordet , W. Leitner , Angew. Chem., Int. Ed. 2023, 62, 202301956. 10.1002/anie.202301956 37345624

[8] H. Chen , J. Yang , J. Su , M. Salmeron , Top. Catal. 2024, 67, 873.

[9] B. Das , A. Thapper , S. Ott , S. B. Colbran , Sustainable Energy Fuels 2019, 3, 2159.

[10] J. M. Le , K. L. Bren , ACS Energy Lett. 2019, 4, 2168.

[11] H.‐L. Wu , X.‐B. Li , C.‐H. Tung , L.‐Z. Wu , Chem. Commun. 2020, 56, 15496. 10.1039/d0cc05870j 33300513

[12] A. Kumar , P. Daw , D. Milstein , Chem. Rev. 2022, 122, 385. 34727501 10.1021/acs.chemrev.1c00412 PMC8759071

[13] A. I. Osman , A. Ayati , P. Krivoshapkin , B. Tanhaei , M. Farghali , P.‐S. Yap , A. Abdelhaleem , Coord. Chem. Rev. 2024, 514, 215900.

[14] B. Limburg , E. Bouwman , S. Bonnet , Coord. Chem. Rev. 2012, 256, 1451.

[15] M. D. Kärkäs , O. Verho , E. V. Johnston , B. Åkermark , Chem. Rev. 2014, 114, 11863. 25354019 10.1021/cr400572f

[16] J. D. Blakemore , R. H. Crabtree , G. W. Brudvig , Chem. Rev. 2015, 115, 12974. 26151088 10.1021/acs.chemrev.5b00122

[17] M. D. Kärkäs , B. Åkermark , Dalton Trans. 2016, 45, 14421. 27142095 10.1039/c6dt00809g

[18] P. L. Dunn , B. J. Cook , S. I. Johnson , A. M. Appel , R. M. Bullock , J. Am. Chem. Soc. 2020, 142, 17845. 32977718 10.1021/jacs.0c08269

[19] H.‐Y. Liu , H. M. C. Lant , C. C. Cody , J. Jelušić , R. H. Crabtree , G. W. Brudvig , ACS Catal. 2023, 13, 4675.

[20] Y. Tian , Z. Mao , L. Wang , J. Liang , Small Struct. 2023, 4, 2200266.

[21] E. E. Benson , C. P. Kubiak , A. J. Sathrum , J. M. Smieja , Chem. Soc. Rev. 2009, 38, 89. 19088968 10.1039/b804323j

[22] R. Francke , B. Schille , M. Roemelt , Chem. Rev. 2018, 118, 4631. 29319300 10.1021/acs.chemrev.7b00459

[23] S. A. Fors , C. A. Malapit , ACS Catal. 2023, 13, 4231.

[24] M. L. Pegis , C. F. Wise , D. J. Martin , J. M. Mayer , Chem. Rev. 2018, 118, 2340. 29406708 10.1021/acs.chemrev.7b00542

[25] C. W. Machan , ACS Catal. 2020, 10, 2640.

[26] S. Dey , B. Mondal , S. Chatterjee , A. Rana , S. Amanullah , A. Dey , Nat. Rev. Chem. 2017, 1, 0098.

[27] H. Liu , L. Wei , F. Liu , Z. Pei , J. Shi , Z.‐j. Wang , D. He , Y. Chen , ACS Catal. 2019, 9, 5245.

[28] M. J. Chalkley , M. W. Drover , J. C. Peters , Chem. Rev. 2020, 120, 5582. 32352271 10.1021/acs.chemrev.9b00638 PMC7493999

[29] N. Hazari , Chem. Soc. Rev. 2010, 39, 4044. 20571678 10.1039/b919680n

[30] Y. Li , H. Wang , C. Priest , S. Li , P. Xu , G. Wu , Adv. Mater. 2021, 33, 2000381. 10.1002/adma.202000381 32671924

[31] M. Yan , Y. Kawamata , P. S. Baran , Chem. Rev. 2017, 117, 13230. 28991454 10.1021/acs.chemrev.7b00397 PMC5786875

[32] N. Kaeffer , W. Leitner , JACS Au 2022, 2, 1266. 35783173 10.1021/jacsau.2c00031 PMC9241009

[33] A. W. Nichols , C. W. Machan , Front. Chem. 2019, 7, 397. 31263689 10.3389/fchem.2019.00397 PMC6584898

[34] R. Matheu , M. Z. Ertem , C. Gimbert‐Suriñach , X. Sala , A. Llobet , Chem. Rev. 2019, 119, 3453. 30816700 10.1021/acs.chemrev.8b00537

[35] I. L. Zak , S. C. Gadekar , A. Milo , Synlett 2021, 32, 329.

[36] J. Trouvé , R. Gramage‐Doria , Chem. Soc. Rev. 2021, 50, 3565. 33502404 10.1039/d0cs01339k

[37] R. M. Bullock , A. Dey , Chem. Rev. 2022, 122, 11897. 35892196 10.1021/acs.chemrev.2c00428

[38] W. Liu , P. J. Das , H. M. Colquhoun , J. F. Stoddart , CCS Chem 2022, 4, 755.

[39] M. W. Drover , Chem. Soc. Rev. 2022, 51, 1861. 35188514 10.1039/d2cs00022a

[40] S. Sinha , C. K. Williams , J. J. Jiang , iScience 2022, 25, 103628. 35005563 10.1016/j.isci.2021.103628 PMC8718893

[41] J. M. Blacquiere , ACS Catal. 2021, 11, 5416.

[42] J. Piera , J.‐E. Bäckvall , Angew. Chem., Int. Ed. 2008, 47, 3506. 10.1002/anie.200700604 18383499

[43] A. Badalyan , S. S. Stahl , Nature 2016, 535, 406. 27350245 10.1038/nature18008

[44] F. Wang , S. S. Stahl , Acc. Chem. Res. 2020, 53, 561. 32049487 10.1021/acs.accounts.9b00544 PMC7295176

[45] W. Shao , B. Lu , J. Cao , J. Zhang , H. Cao , F. Zhang , C. Zhang , Chem Asian J. 2023, 18, 202201093. 10.1002/asia.202201093 36577711

[46] A. G. Reid , C. W. Machan , J. Am. Chem. Soc. 2023, 145, 2013. 36652254 10.1021/jacs.2c10033

[47] J.‐M. Savéant , Angew. Chem., Int. Ed. 2019, 58, 2125.

[48] Z. Thammavongsy , I. P. Mercer , J. Y. Yang , Chem. Commun. 2019, 55, 10342. 10.1039/c9cc05139b 31424056

[49] A. C. Ghosh , C. Duboc , M. Gennari , Coord. Chem. Rev. 2021, 428, 213606.

[50] H.‐C. Ma , S.‐C. Hsiao , Y.‐H. Wang , Catal. Sci. Technol. 2023, 13, 1598.

[51] M. Haake , B. Reuillard , M. Chavarot‐Kerlidou , C. Costentin , V. Artero , Angew. Chem., Int. Ed. 2024, 63, 202413910. 10.1002/anie.202413910 39555743

[52] J. L. Dempsey , Acc. Chem. Res. 2025, 58, 947. 40036191 10.1021/acs.accounts.5c00002

[53] R. Francke , R. D. Little , Chem. Soc. Rev. 2014, 43, 2492. 24500279 10.1039/c3cs60464k

[54] A. Ghosh , S. Dasgupta , A. Kundu , S. Mandal , Dalton Trans. 2022, 51, 10320. 35730327 10.1039/d2dt01124g

[55] W. Zhang , W. Lai , R. Cao , Chem. Rev. 2017, 117, 3717. 28222601 10.1021/acs.chemrev.6b00299

[56] E. Boutin , L. Merakeb , B. Ma , B. Boudy , M. Wang , J. Bonin , E. Anxolabéhère‐Mallart , M. Robert , Chem. Soc. Rev. 2020, 49, 5772. 10.1039/d0cs00218f 32697210

[57] K. J. Lee , K. M. Lodaya , C. T. Gruninger , E. S. Rountree , J. L. Dempsey , Chem. Sci. 2020, 11, 9836. 34094244 10.1039/d0sc02592e PMC8162168

[58] C. M. Harvey , C. Costentin , ACS Catal. 2025, 15, 14927.

[59] P. R. Wells , Chem. Rev. 1963, 63, 171.

[60] C. Costentin , J.‐M. Savéant , Nat. Rev. Chem. 2017, 1, 0087.

[61] B. M. Stratakes , J. L. Dempsey , A. J. M. Miller , ChemElectroChem 2021, 8, 4161.

[62] D. R. Weinberg , C. J. Gagliardi , J. F. Hull , C. F. Murphy , C. A. Kent , B. C. Westlake , A. Paul , D. H. Ess , D. G. McCafferty , T. J. Meyer , Chem. Rev. 2012, 112, 4016. 22702235 10.1021/cr200177j

[63] S. Hammes‐Schiffer , Energy Environ. Sci. 2012, 5, 7696.

[64] R. Tyburski , T. Liu , S. D. Glover , L. Hammarström , J. Am. Chem. Soc. 2021, 143, 560. 33405896 10.1021/jacs.0c09106 PMC7880575

[65] D. G. Nocera , J. Am. Chem. Soc. 2022, 144, 1069. 35023740 10.1021/jacs.1c10444

[66] C. Costentin , M. Robert , J.‐M. Savéant , Acc. Chem. Res. 2010, 43, 1019. 20232879 10.1021/ar9002812

[67] J.‐M. Savéant , Annu. Rev. Anal. Chem. 2014, 7, 537. 10.1146/annurev-anchem-071213-020315 25014349

[68] G. A. Parada , Z. K. Goldsmith , S. Kolmar , B. Pettersson Rimgard , B. Q. Mercado , L. Hammarström , S. Hammes‐Schiffer , J. M. Mayer , Science 2019, 364, 471. 30975771 10.1126/science.aaw4675 PMC6681808

[69] R. G. Agarwal , S. C. Coste , B. D. Groff , A. M. Heuer , H. Noh , G. A. Parada , C. F. Wise , E. M. Nichols , J. J. Warren , J. M. Mayer , Chem. Rev. 2022, 122, 1. 34928136 10.1021/acs.chemrev.1c00521 PMC9175307

[70] P. R. D. Murray , J. H. Cox , N. D. Chiappini , C. B. Roos , E. A. McLoughlin , B. G. Hejna , S. T. Nguyen , H. H. Ripberger , J. M. Ganley , E. Tsui , N. Y. Shin , B. Koronkiewicz , G. Qiu , R. R. Knowles , Chem. Rev. 2022, 122, 2017. 34813277 10.1021/acs.chemrev.1c00374 PMC8796287

[71] N. E. S. Tay , D. Lehnherr , T. Rovis , Chem. Rev. 2022, 122, 2487. 34751568 10.1021/acs.chemrev.1c00384 PMC10021920

[72] F. F. Khan , A. D. Chowdhury , G. K. Lahiri , Eur. J. Inorg. Chem. 2020, 2020, 1138.

[73] B. Mondal , S. Ye , Coord. Chem. Rev. 2020, 405, 213115.

[74] B. Singh , A. Indra , Inorg. Chim. Acta 2020, 506, 119440.

[75] M. Sutradhar , A. J. L. Pombeiro , J. A. L. da Silva , Coord. Chem. Rev. 2021, 439, 213911.

[76] A. Nakada , T. Matsumoto , H.‐C. Chang , Coord. Chem. Rev. 2022, 473, 214804.

[77] A. Migliore , N. F. Polizzi , M. J. Therien , D. N. Beratan , Chem. Rev. 2014, 114, 3381. 24684625 10.1021/cr4006654 PMC4317057

[78] V. R. I. Kaila , Acc. Chem. Res. 2021, 54, 4462. 34894649 10.1021/acs.accounts.1c00524 PMC8697550

[79] S. Raugei , D. L. DuBois , R. Rousseau , S. Chen , M.‐H. Ho , R. M. Bullock , M. Dupuis , Acc. Chem. Res. 2015, 48, 248. 25574854 10.1021/ar500342g

[80] M. J. Chalkley , P. Garrido‐Barros , J. C. Peters , Science 2020, 369, 850. 32792399 10.1126/science.abc1607

[81] L. Cardinale , S. S. Stahl , D. Kalyani , D. Lehnherr , Adv. Catal. 2023, 72, 57.

[82] M. Garland , in Encyclopedia of Catalysis, Wiley, New Jersey 2010.

[83] K. Grabow , U. Bentrup , ACS Catal. 2014, 4, 2153.

[84] K. Köhnke , N. Wessel , J. Esteban , J. Jin , A. J. Vorholt , W. Leitner , Green Chem. 2022, 24, 1951.

[85] D. L. DuBois , Inorg. Chem. 2014, 53, 3935. 24555579 10.1021/ic4026969

[86] B. H. Solis , S. Hammes‐Schiffer , Inorg. Chem. 2014, 53, 6427. 24731018 10.1021/ic5002896

[87] X. Wu , F. Li , B. Zhang , L. Sun , J. Photochem. Photobiol., C. 2015, 25, 71.

[88] K. J. Lee , B. D. McCarthy , J. L. Dempsey , Chem. Soc. Rev. 2019, 48, 2927. 31089606 10.1039/c8cs00851e

[89] H. Kisch , D. Bahnemann , J. Phys. Chem. Lett. 2015, 6, 1907. 26263267 10.1021/acs.jpclett.5b00521

[90] P. A. Kempler , A. C. Nielander , Nat. Commun. 2023, 14, 1158. 36859528 10.1038/s41467-023-36880-8 PMC9977834

[91] N. Dutta , D. Bagchi , G. Chawla , S. C. Peter , ACS Energy Lett. 2024, 9, 323.

[92] J. Rabani , H. Mamane , D. Pousty , J. R. Bolton , Photochem. Photobiol. 2021, 97, 873. 34124787 10.1111/php.13429

[93] H. Kuhn , S. Braslavsky , R. Schmidt , Pure Appl. Chem. 2004, 76, 2105.

[94] Y. Ji , D. A. DiRocco , C. M. Hong , M. K. Wismer , M. Reibarkh , Org. Lett. 2018, 20, 2156. 29589943 10.1021/acs.orglett.8b00391

[95] E. Stadler , A. Eibel , D. Fast , H. Freißmuth , C. Holly , M. Wiech , N. Moszner , G. Gescheidt , Photochem. Photobiol. Sci. 2018, 17, 660. 29714365 10.1039/c7pp00401j

[96] Z. Wang , C. Li , K. Domen , Chem. Soc. Rev. 2019, 48, 2109. 30328438 10.1039/c8cs00542g

[97] Y.‐G. Yu , G. Chen , L.‐X. Hao , Y.‐S. Zhou , Y. Wang , J. Pei , J.‐X. Sun , Z.‐H. Han , Chem. Commun. 2013, 49, 10142. 10.1039/c3cc45568h 24048349

[98] F. Mohamadpour , A. M. Amani , RSC Adv. 2024, 14, 20609. 38952944 10.1039/d4ra03259d PMC11215501

[99] S. B. Beil , S. Bonnet , C. Casadevall , R. J. Detz , F. Eisenreich , S. D. Glover , C. Kerzig , L. Næsborg , S. Pullen , G. Storch , N. Wei , C. Zeymer , JACS Au 2024, 4, 2746. 39211583 10.1021/jacsau.4c00527 PMC11350580

[100] M. A. Cismesia , T. P. Yoon , Chem. Sci. 2015, 6, 5426. 26668708 10.1039/c5sc02185e PMC4676763

[101] C. Costentin , S. Drouet , M. Robert , J.‐M. Savéant , J. Am. Chem. Soc. 2012, 134, 11235. 22670885 10.1021/ja303560c

[102] S. Kozuch , J. M. L. Martin , ACS Catal. 2012, 2, 2787.

[103] S. Anantharaj , P. E. Karthik , S. Noda , Angew. Chem., Int. Ed. 2021, 60, 23051. 10.1002/anie.202110352 PMC8596788 34523770

[104] E. S. Rountree , B. D. McCarthy , T. T. Eisenhart , J. L. Dempsey , Inorg. Chem. 2014, 53, 9983. 25247280 10.1021/ic500658x

[105] C. Costentin , J.‐M. Savéant , ChemElectroChem 2014, 1, 1226.

[106] G. Graziono , Nat. Rev. Chem. 2018, 2, 0130.

[107] W.‐C. Hsu , Y.‐H. Wang , ChemSusChem 2022, 15, 202102378.

[108] I. Azcarate , C. Costentin , M. Robert , J.‐M. Savéant , J. Am. Chem. Soc. 2016, 138, 16639. 27976580 10.1021/jacs.6b07014

[109] T. Z. H. Gani , H. J. Kulik , ACS Catal. 2018, 8, 975.

[110] D. J. Martin , C. F. Wise , M. L. Pegis , J. M. Mayer , Acc. Chem. Res. 2020, 53, 1056. 32281786 10.1021/acs.accounts.0c00044 PMC7351079

[111] W. Nie , C. C. L. McCrory , Dalton Trans. 2022, 51, 6993. 35383803 10.1039/d2dt00333c

[112] E. V. D. Anslyn , A. Dennis , Modern Physical Organic Chemistry, 1st ed., University Science Books, Sausalito, CA, 2006.

[113] C. B. Santiago , J.‐Y. Guo , M. S. Sigman , Chem. Sci. 2018, 9, 2398. 29719711 10.1039/c7sc04679k PMC5903422

[114] Y.‐H. Wang , M. L. Pegis , J. M. Mayer , S. S. Stahl , J. Am. Chem. Soc. 2017, 139, 16458. 29039921 10.1021/jacs.7b09089

[115] Y.‐H. Wang , B. Mondal , S. S. Stahl , ACS Catal. 2020, 10, 12031.

[116] S. Sinha , A. Chaturvedi , N. Devi , P. Tiwari , J. Jiang , J. Chem. Ed. 2025, 102, 415.

[117] P. T. Smith , S. Weng , C. J. Chang , Inorg. Chem. 2020, 59, 9270. 32623894 10.1021/acs.inorgchem.0c01162

[118] S. Dey , F. Masero , E. Brack , M. Fontecave , V. Mougel , Nature 2022, 607, 499. 35859199 10.1038/s41586-022-04874-z

[119] S. L. Hooe , J. J. Moreno , A. G. Reid , E. N. Cook , C. W. Machan , Angew. Chem., Int. Ed. 2022, 61, 202109645. 10.1002/anie.202109645 34695281

[120] A. G. Reid , J. J. Moreno , S. L. Hooe , K. R. Baugh , I. H. Thomas , D. A. Dickie , C. W. Machan , Chem. Sci. 2022, 13, 9595. 36091894 10.1039/d2sc03258a PMC9400620

[121] A. G. Reid , M. E. Moberg , C. A. Koellner , J. J. Moreno , S. L. Hooe , K. R. Baugh , D. A. Dickie , C. W. Machan , Organometallics 2023, 42, 1139.

[122] A. G. Reid , E. A. Zelenke , M. E. Moberg , D. A. Dickie , C. W. Machan , Chem. Commun. 2024, 60, 8208. 10.1039/d4cc01988a 39015067

[123] B. X. Nguyen , X. Li , J. J. Warren , ACS Catal. 2024, 14, 12260.

[124] A. Sonea , K. L. Branch , J. J. Warren , ACS Catal. 2023, 13, 3902.

[125] A. Sonea , N. R. Crudo , J. J. Warren , J. Am. Chem. Soc. 2024, 146, 3721. 38307036 10.1021/jacs.3c10127

[126] A. Sonea , J. J. Warren , ACS Catal. 2025, 15, 1444.

[127] M. E. Moberg , A. G. Reid , D. A. Dickie , C. W. Machan , Dalton Trans. 2024, 53, 16849. 39189075 10.1039/d4dt01981d

[128] S. Dinda , A. I. Siddiqui , S. Behera , B. Mondal , ACS Catal. 2023, 13, 12643.

[129] C. W. Anson , S. S. Stahl , J. Am. Chem. Soc. 2017, 139, 18472. 29198114 10.1021/jacs.7b11362

[130] C. W. Anson , S. Ghosh , S. Hammes‐Schiffer , S. S. Stahl , J. Am. Chem. Soc. 2016, 138, 4186. 26924338 10.1021/jacs.6b00254

[131] B. Sun , Z. Ou , D. Meng , Y. Fang , Y. Song , W. Zhu , P. V. Solntsev , V. N. Nemykin , K. M. Kadish , Inorg. Chem. 2014, 53, 8600. 25068447 10.1021/ic501210t

[132] S. N. Chowdhury , S. Biswas , P. Das , S. Paul , A. N. Biswas , Inorg. Chem. 2020, 59, 14012. 32916051 10.1021/acs.inorgchem.0c01776

[133] A. Das , A. Ali , G. Gupta , A. Santra , P. Jain , P. P. Ingole , S. Paul , S. Paria , ACS Catal. 2023, 13, 5285.

[134] A. Santra , A. Das , S. Kaur , P. Jain , P. P. Ingole , S. Paria , Chem. Sci. 2024, 15, 4095. 38487234 10.1039/d3sc06753j PMC10935699

[135] A. Das , A. Santra , A. Kumari , D. Ghosh , S. Paria , J. Am. Chem. Soc. 2025, 147, 6549. 39946092 10.1021/jacs.4c14877

[136] C. T. Carver , B. D. Matson , J. M. Mayer , J. Am. Chem. Soc. 2012, 134, 5444. 22394189 10.1021/ja211987f

[137] M. L. Pegis , B. A. McKeown , N. Kumar , K. Lang , D. J. Wasylenko , X. P. Zhang , S. Raugei , J. M. Mayer , ACS Cent. Sci. 2016, 2, 850. 27924314 10.1021/acscentsci.6b00261 PMC5126711

[138] S. Bhunia , A. Rana , P. Roy , D. J. Martin , M. L. Pegis , B. Roy , A. Dey , J. Am. Chem. Soc. 2018, 140, 9444. 29975839 10.1021/jacs.8b02983

[139] S. Bhunia , A. Ghatak , A. Rana , A. Dey , J. Am. Chem. Soc. 2023, 145, 3812. 36744304 10.1021/jacs.2c13552

[140] S. V. Obisesan , C. Rose , B. H. Farnum , C. R. Goldsmith , J. Am. Chem. Soc. 2022, 144, 22826. 36493464 10.1021/jacs.2c08315

[141] S. V. Obisesan , M. Parvin , M. Tao , E. Ramos , A. C. Saunders , B. H. Farnum , C. R. Goldsmith , Inorg. Chem. 2024, 63, 14126. 39008564 10.1021/acs.inorgchem.4c01977

[142] A. Ali , D. Prakash , P. Majumder , S. Ghosh , A. Dutta , ACS Catal. 2021, 11, 5934.

[143] A. Ali , D. Prakash , A. Saini , C. Das , N. A. Shah , A. Dutta , ChemCatChem 2025, 17, 202401228.

[144] S. W. Gersten , G. J. Samuels , T. J. Meyer , J. Am. Chem. Soc. 1982, 104, 4029.

[145] J. A. Gilbert , D. S. Eggleston , W. R. Murphy , D. A. Geselowitz , S. W. Gersten , D. J. Hodgson , T. J. Meyer , J. Am. Chem. Soc. 1985, 107, 3855.

[146] J. J. Concepcion , J. W. Jurss , J. L. Templeton , T. J. Meyer , Proc. Natl. Acad. Sci. USA 2008, 105, 17632. 19004763 10.1073/pnas.0807153105 PMC2584677

[147] M. V. Sheridan , B. D. Sherman , Z. Fang , K.‐R. Wee , M. K. Coggins , T. J. Meyer , ACS Catal. 2015, 5, 4404.

[148] J. J. Concepcion , J. W. Jurss , P. G. Hoertz , T. J. Meyer , Angew. Chem., Int. Ed. 2009, 48, 9473. 10.1002/anie.200901279 19904782

[149] J. J. Concepcion , J. W. Jurss , J. L. Templeton , T. J. Meyer , J. Am. Chem. Soc. 2008, 130, 16462. 19554681 10.1021/ja8059649

[150] M. R. Norris , J. J. Concepcion , D. P. Harrison , R. A. Binstead , D. L. Ashford , Z. Fang , J. L. Templeton , T. J. Meyer , J. Am. Chem. Soc. 2013, 135, 2080. 23336109 10.1021/ja311645d

[151] D. M. Ryan , M. K. Coggins , J. J. Concepcion , D. L. Ashford , Z. Fang , L. Alibabaei , D. Ma , T. J. Meyer , M. L. Waters , Inorg. Chem. 2014, 53, 8120. 25046035 10.1021/ic5011488

[152] F. Liu , J. J. Concepcion , J. W. Jurss , T. Cardolaccia , J. L. Templeton , T. J. Meyer , Inorg. Chem. 2008, 47, 1727. 18330966 10.1021/ic701249s

[153] J. J. Concepcion , J. W. Jurss , M. R. Norris , Z. F. Chen , J. L. Templeton , T. J. Meyer , Inorg. Chem. 2010, 49, 1277. 20058918 10.1021/ic901437e

[154] J. J. Concepcion , M.‐K. Tsai , J. T. Muckerman , T. J. Meyer , J. Am. Chem. Soc. 2010, 132, 1545. 20085264 10.1021/ja904906v

[155] M. R. Norris , J. J. Concepcion , Z. Fang , J. L. Templeton , T. J. Meyer , Angew. Chem., Int. Ed. 2013, 52, 13580. 10.1002/anie.201305951 24346943

[156] N. Kaveevivitchai , R. Chitta , R. Zong , M. El Ojaimi , R. P. Thummel , J. Am. Chem. Soc. 2012, 134, 10721. 22698450 10.1021/ja300797g

[157] L. Kohler , N. Kaveevivitchai , R. Zong , R. P. Thummel , Inorg. Chem. 2014, 53, 912. 24364791 10.1021/ic4022905

[158] H. N. Kagalwala , L. Tong , R. Zong , L. Kohler , M. S. G. Ahlquist , T. Fan , K. J. Gagnon , R. P. Thummel , ACS Catal. 2017, 7, 2607.

[159] A. G. Nash , C. J. Breyer , B. D. Vincenzini , G. I. Elliott , J. Niklas , O. G. Poluektov , A. L. Rheingold , D. K. Smith , D. G. Musaev , D. B. Grotjahn , Angew. Chem., Int. Ed. 2021, 60, 1540. 10.1002/anie.202008896 32966708

[160] N. Kaveevivitchai , R. Zong , H.‐W. Tseng , R. Chitta , R. P. Thummel , Inorg. Chem. 2012, 51, 2930. 22339431 10.1021/ic202174j

[161] B. Zhang , L. Sun , J. Am. Chem. Soc. 2019, 141, 5565. 30889353 10.1021/jacs.8b12862

[162] R. Matheu , M. Z. Ertem , J. Benet‐Buchholz , E. Coronado , V. S. Batista , X. Sala , A. Llobet , J. Am. Chem. Soc. 2015, 137, 10786. 26226390 10.1021/jacs.5b06541

[163] N. Vereshchuk , R. Matheu , J. Benet‐Buchholz , M. Pipelier , J. Lebreton , D. Dubreuil , A. Tessier , C. Gimbert‐Suriñach , M. Z. Ertem , A. Llobet , J. Am. Chem. Soc. 2020, 142, 5068. 32045521 10.1021/jacs.9b11935

[164] T. Liu , S. Zhan , N. Shen , L. Wang , Z. Szabó , H. Yang , M. S. G. Ahlquist , L. Sun , J. Am. Chem. Soc. 2023, 145, 11818. 37196315 10.1021/jacs.3c03415 PMC10236490

[165] L. Wang , L. Duan , B. Stewart , M. Pu , J. Liu , T. Privalov , L. Sun , J. Am. Chem. Soc. 2012, 134, 18868. 23062211 10.1021/ja309805m

[166] J. Yang , L. Wang , S. Zhan , H. Zou , H. Chen , M. S. G. Ahlquist , L. Duan , L. Sun , Nat. Commun. 2021, 12, 373. 33446649 10.1038/s41467-020-20637-8 PMC7809030

[167] R. Matheu , M. Z. Ertem , C. Gimbert‐Suriñach , J. Benet‐Buchholz , X. Sala , A. Llobet , ACS Catal. 2017, 7, 6525.

[168] L. Duan , A. Fischer , Y. Xu , L. Sun , J. Am. Chem. Soc. 2009, 131, 10397. 19601625 10.1021/ja9034686

[169] P. Garrido‐Barros , J. Derosa , M. J. Chalkley , J. C. Peters , Nature 2022, 609, 71. 36045240 10.1038/s41586-022-05011-6 PMC10281199

[170] E. A. McLoughlin , K. C. Armstrong , R. M. Waymouth , ACS Catal. 2020, 10, 11654.

[171] C. M. Galvin , R. M. Waymouth , J. Am. Chem. Soc. 2020, 142, 19368. 33138365 10.1021/jacs.0c09605

[172] J. Liu , A. Guðmundsson , J.‐E. Bäckvall , Angew. Chem., Int. Ed. 2021, 60, 15686. 10.1002/anie.202012707 PMC9545650 33368909

[173] G. Csjernyik , A. H. Éll , L. Fadini , B. Pugin , J.‐E. Bäckvall , J. Org. Chem. 2002, 67, 1657. 11871899 10.1021/jo0163750

[174] E. V. Johnston , E. A. Karlsson , L. H. Tran , B. Åkermark , J. E. Bäckvall , Eur. J. Org. Chem. 2010, 2010, 1971.

[175] B. W. Purse , L.‐H. Tran , J. Piera , B. Åkermark , J.‐E. Bäckvall , Chem. ‐ Eur. J. 2008, 14, 7500. 18604855 10.1002/chem.200800657

[176] C. Zhu , J. Liu , B. K. Mai , F. Himo , J.‐E. Bäckvall , J. Am. Chem. Soc. 2020, 142, 5751. 32101690 10.1021/jacs.9b13700 PMC7307908

[177] J. Liu , J.‐E. Bäckvall , Chem.‐Eur. J. 2020, 26, 15513.32960479

[178] A. Guðmundsson , K. E. Schlipköter , J.‐E. Bäckvall , Angew. Chem., Int. Ed. 2020, 59, 5403. 10.1002/anie.202000054 PMC7154773 31999013

[179] A. Guðmundsson , S. Manna , J.‐E. Bäckvall , Angew. Chem., Int. Ed. 2021, 60, 11819. 10.1002/anie.202102681 PMC8252094 33725364

[180] T. Gupta , S. Yati , A. Mondal , B. Mondal , ACS Catal. 2024, 14, 17690.

[181] J. Derosa , P. Garrido‐Barros , J. C. Peters , J. Am. Chem. Soc. 2021, 143, 9303. 34138550 10.1021/jacs.1c03335

[182] C. J. Gagliardi , B. C. Westlake , C. A. Kent , J. J. Paul , J. M. Papanikolas , T. J. Meyer , Coord. Chem. Rev. 2010, 254, 2459.

[183] O. S. Wenger , Chem.‐Eur. J. 2011, 17, 11692. 21905142 10.1002/chem.201102011

[184] J. C. Lennox , D. A. Kurtz , T. Huang , J. L. Dempsey , ACS Energy Lett. 2017, 2, 1246.

[185] P. Garrido‐Barros , C. G. Romero , J. R. Winkler , J. C. Peters , J. Am. Chem. Soc. 2024, 146, 12750. 38669102 10.1021/jacs.4c02610 PMC11082884

